# Profiles of environmental sensitivity among nursing students on clinical placement and their structural associations with failure mindsets: a latent profile and network analysis

**DOI:** 10.3389/fpsyg.2026.1865123

**Published:** 2026-08-12

**Authors:** Jialin Lv, Lei Wang, Minle Lu, Yuan Zhao, Junhui Dong, Lixiu Zhang, Ying Wang, Peimin Lin

**Affiliations:** 1 Department of Nursing, School of Medicine, Huzhou Normal University, Huzhou, Zhejiang, China; 2 Department of Nursing, School of Medicine, Shandong University of Traditional Chinese Medicine, Jinan, Shandong, China; 3 The First Medical Center of Chinese PLA General Hospital, Beijing, China; 4 Joint Shantou International Eye Center of Shantou University and the Chinese University of Hong Kong, Shantou, Guangdong, China; 5 Fifth Clinical Institute of Shantou University Medical College, Shantou, Guangdong, China

**Keywords:** clinical placement, environmental sensitivity, failure mindset, latent profile analysis, network analysis, nursing students

## Abstract

**Aim:**

To identify latent profiles of environmental sensitivity among nursing students on clinical placement and examine their structural associations with failure mindsets.

**Background:**

Environmental sensitivity shapes how individuals process stimuli, interacting with failure experiences during nursing clinical placements to influence professional adaptation. Grounded in the trait-stress model and Differential Susceptibility Theory, we hypothesised that distinct sensitivity profiles would differentially shape failure mindsets. However, heterogeneity within this trait and its links to failure cognition remain underexplored.

**Design:**

A cross-sectional survey design was employed.

**Methods:**

Latent Profile Analysis (LPA) was conducted using the College Students’ Environmental Sensitivity Scale, with model fit evaluated using *BIC*, *SABIC*, and the Bootstrap Likelihood Ratio Test (*BLRT*). Multivariate logistic regression identified factors influencing subgroup membership. Network stability was assessed via case-dropping subset bootstrapping (CS-coefficient). Network analysis formally compared the structural associations between environmental sensitivity dimensions and failure mindset components across subgroups using the Network Comparison Test (NCT) with 10,000 permutations.

**Results:**

Three distinct profiles emerged: Highly Sensitive-Reflective (12.89%), Balanced-Adaptive (65.78%), and Low Sensitive-Rational (21.33%). The Highly Sensitive-Reflective subgroup exhibited the highest negative failure mindset, whilst the Low Sensitive-Rational subgroup scored highest on positive failure mindset. Network analysis revealed that emotional reactivity served as the core node in the Highly Sensitive subgroup, with strong negative coupling between positive and negative failure mindsets. In the Low Sensitive subgroup, the two failure mindset components were structurally independent. Regression analysis identified professional entry motivation, occupational identity, self-efficacy, gender, educational level, and family income as significant predictors of subgroup membership.

**Conclusion:**

Environmental sensitivity among nursing students on clinical placement is heterogeneous and meaningfully associated with a failure mindset. These findings suggest the potential utility of stratified, typology-based clinical placement management approaches that incorporate targeted support strategies to reduce the risk of negative failure cognitions and promote professional sustainability.

## 1 Introduction

The global nursing staff shortage has become a major public health issue, threatening the sustainable operation of healthcare systems. The World Health Organisation (WHO) predicts that by 2030, the global nursing shortage will reach 5.9 million, with low- and middle-income countries particularly affected (World Health Organization, 2025). In China, as the population ages rapidly and the burden of chronic diseases continues to grow, the contradiction between rising demand for nursing care and the insufficient supply of clinical nurses is becoming increasingly apparent, posing severe challenges to the stability and career continuity of the nursing workforce (Zhang et al., 2025). Nursing placements represent a crucial stage in the transition of nursing students from theoretical learning to clinical practice, and constitute a pivotal period for the formation of professional identity and the development of professional competence (Amin et al., 2026). During this phase, students not only face intensive practical training and complex requirements for doctor-patient communication, but must also complete their professional self-construction amidst ongoing supervisory assessments and discrepancies between role expectations (Yao and Wu, 2025). Previous research indicates that the multiple pressures and frequent failure scenarios arising from clinical placements are significant contributors to trainees’ anxiety, depression, and low professional identity, and are closely linked to the attrition of nursing talent (Chang et al., 2026). However, when confronted with the same clinical stressors, there are significant differences in psychological coping patterns among individuals, suggesting that personal traits play a crucial moderating role in the perception and adaptation to stress (Gong et al., 2025).

Environmental sensitivity refers to a personality trait characterised by an individual’s perception, registration, and in-depth processing of environmental stimuli, encompassing four core dimensions: Deep perception, Deep processing of information, Proneness to overstimulation, and High emotional reactivity (Gubler et al., 2025). The present study conceptualises environmental sensitivity within the established tradition of Sensory Processing Sensitivity (SPS), a biologically-grounded trait characterising individuals who process environmental stimuli with greater depth, commonly termed the Highly Sensitive Person (HSP). SPS is empirically operationalised via the DOES model: Depth of processing, Overstimulation proneness, Emotional reactivity/Empathy, and Sensitivity to subtleties, corresponding closely to the four dimensions assessed in the present study. Whilst environmental sensitivity and high sensitivity are used interchangeably as general descriptors of this trait, the term environmental susceptibility is specifically reserved for Differential Susceptibility Theory (DST) contexts, where bidirectional plasticity across both adverse and supportive environments is explicitly emphasised. This trait operates bidirectionally. For nursing students, high environmental sensitivity endows them with keener clinical observation skills and greater empathy, helping them to identify patients’ subtle needs and build high-quality nurse–patient relationships (Lionetti and Pluess, 2024); however, in high-pressure clinical settings, highly sensitive individuals are also more prone to over-activation in response to negative environmental cues, leading to emotional exhaustion and cognitive overload (Li et al., 2024). Specifically, clinical wards expose nursing students to dense sensory and emotional demands, including alarm noise, rapid task switching, complex technical procedures, patient suffering, time pressure, and continuous performance evaluation (Gaberson et al., 2024). For students with a high propensity for overstimulation and emotional reactivity, these stimuli may intensify cognitive load and emotional arousal, thereby affecting their confidence, technical performance, and responses to clinical errors (Ponce-Valencia et al., 2024). According to Differential Susceptibility Theory and the Orchid-Dandelion model, individuals with higher environmental sensitivity are more responsive to both negative and positive environmental stimuli (Assary et al., 2023; Ellis and Boyce, 2008). In clinical nursing education, highly sensitive students may therefore process supervisor criticism, procedural errors, or negative feedback more intensely than their less sensitive peers. This heightened responsiveness may make clinical setbacks more likely to be interpreted as personal inadequacy, thereby contributing to a more debilitating mindset regarding failure. Conversely, in supportive teaching environments characterised by constructive feedback, empathic supervision, and opportunities for safe error correction, highly sensitive students may also benefit more strongly from positive guidance and transform failure experiences into reflective learning and professional growth (Butler and Lyman, 2025).

A failure mindset refers to the stable cognitive and evaluative tendencies that an individual develops following experiences of setbacks, comprising two relatively independent dimensions: positive and negative (Sim and Wong, 2010). A positive failure mindset views setbacks as changeable opportunities for growth, driving individuals to reflect on their experiences proactively, optimise their behaviour, and sustain their motivation to learn (Burnette et al., 2023); a negative failure mindset, on the other hand, tends to attribute failure to fixed, unchangeable deficiencies in one’s own abilities, and is likely to induce self-doubt, avoidance of challenges and learned helplessness (Abramson et al., 1978). In the context of nursing placements, operational errors, criticism from supervisors, and the gap between theory and practice are frequent sources of failure; the way students interpret these experiences directly influences their career development trajectory (Li et al., 2024). Those with a positive mindset towards failure can extract lessons from repeated setbacks, build clinical competence, and sustain their drive for professional exploration (Williams, 2021); conversely, a negative mindset may further amplify the perception of negative stimuli, exacerbate anxiety and confusion, and ultimately lead to professional burnout or even a tendency to leave the profession (Zheng et al., 2024).

The “trait-stress model” provides an important theoretical foundation for the intrinsic relationship between the two; it posits that there is an interactive effect between an individual’s internal susceptibility traits and external environmental stressors (Zubin and Spring, 1977; Nhem et al., 2026). High environmental sensitivity, as a risk-susceptibility trait, makes individuals more vulnerable to adverse effects in negative situations (Aron and Aron, 1997). In the context of nursing placements, high-intensity clinical demands, persistent assessment pressures, and frequent experiences of failure collectively constitute significant external stressors (Zhang et al., 2026). Furthermore, the heightened perception and detailed processing of these stimuli by individuals with high environmental sensitivity may further reinforce their negative cognitive evaluations of failure, making it easier for a negative mindset toward failure to be activated and entrenched (Pluess, 2015). In other words, environmental sensitivity does not influence an individual’s psychological state in isolation; its effects are likely to act indirectly on professional adaptation outcomes by shaping the individual’s cognitive interpretative framework of failure situations (Pluess, 2015). Building on the trait-stress model, Differential Susceptibility Theory further emphasises the bidirectional plasticity of environmental sensitivity (Belsky and Pluess, 2009). Individuals with high environmental susceptibility may be more vulnerable under adverse clinical conditions but may also benefit more from supportive teaching environments. This theoretical perspective suggests that accurately identifying high-sensitivity subgroups and providing positive mentoring, constructive feedback, and psychological support may help transform their perceptual depth and empathic potential into professional strengths (Atta et al., 2024). However, most current studies treat environmental sensitivity as a single continuous variable (Greven et al., 2019), overlooking its multidimensional and heterogeneous composition within individuals, and thus failing to reflect the true distribution of differences in susceptibility traits among nursing students (Lionetti et al., 2018). Furthermore, existing research has largely focused on the association between environmental sensitivity and emotional outcomes such as anxiety and depression; few studies have directly examined its relationship with a mindset of failure, and there is a particular lack of systematic investigation targeting the specific occupational group of nursing students (Zhang et al., 2026). Therefore, this study utilises Latent Profile Analysis (LPA) to identify heterogeneous subgroups of environmental sensitivity, combines it with multivariate logistic regression to explore factors influencing subgroup membership, and employs network analysis to compare differences in the structural associations between environmental sensitivity and a failure mindset across subgroups. The findings will not only deepen our understanding of individual differences in environmental sensitivity among nursing students but also provide evidence-based grounds for developing targeted psychological interventions and optimising clinical teaching strategies based on subgroup characteristics. Ultimately, this will provide actionable pathways to address the significant public health challenge posed by the global nursing workforce shortage.

Based on the above theoretical framework, three explicit research hypotheses are proposed.

*H1*: Environmental sensitivity among nursing students during clinical placements manifests as heterogeneous latent profiles rather than a homogeneous continuum, reflecting meaningful individual differences in trait expression.

*H2*: Students in the high-sensitivity profile will exhibit significantly greater negative failure mindset scores and lower positive failure mindset scores compared with those in low- or balanced-sensitivity profiles.

*H3*: The network structure of associations between environmental sensitivity dimensions and failure mindset components will differ across profiles, with emotional reactivity expected to occupy a more central position in the high-sensitivity profile.

## 2 Methods

### 2.1 Participants

This study employed a cross-sectional design, utilising convenience sampling to administer a questionnaire survey between June and December 2025 in Zhejiang, Shanxi, Jiangsu, Sichuan, and Fujian provinces in China. These provinces include both developed eastern coastal regions and inland provinces, which helped broaden the geographical coverage and contextual diversity of the sample in terms of nursing education resources and clinical placement environments. The study participants were nursing students currently undertaking clinical placements in hospitals across these provinces. Although the sample size was relatively large, the use of convenience sampling may have introduced selection bias (Stratton, 2021). Therefore, the distribution of latent profiles observed in this study should be interpreted with caution and may not fully represent all nursing students in China. To provide further contextual information, participants were recruited from clinical placement settings across different provincial regions, which may help readers evaluate the applicability and generalisability of the findings. All participating hospitals were public tertiary teaching hospitals affiliated with nursing education programmes in their respective provinces.

The inclusion criteria for participants were: (1) current nursing students undertaking clinical placements in hospitals; (2) those with a placement duration of at least 1 month and some clinical experience; (3) those who provided informed consent and voluntarily participated in this study. The exclusion criteria were: (1) questionnaire completion time of less than 300 s; this threshold was determined following a pilot study and in reference to relevant benchmarks from the literature on careless responses; (2) completion time exceeding 60 min, to exclude records where insufficient effort was expended; (3) providing identical answers to 10 or more consecutive items, which was deemed to constitute pattern-based responses; furthermore, the platform’s technical safeguards prevented duplicate submissions by the same participant, whilst the anonymised design further reduced the likelihood of repeated responses. A total of 1,200 questionnaires were distributed; after the above screening process, 1,125 were recovered, yielding a valid response rate of 93.75%. The final valid sample included in the analysis comprised 1,125 participants, of whom 960 were female (85.33%), and 165 were male (14.67%), with a mean age of (20.73 ± 2.43) years.

The adequacy of the achieved sample size was evaluated across the three primary analytic methods. According to the reference standards for sample size in previous LPA studies (Spurk et al., 2020), when the number of categories does not exceed five and the number of indicators does not exceed six, a sample size of 500 or more is sufficient to ensure the stability of model parameter estimates and classification accuracy; the present sample of 1,125 substantially exceeds this threshold. For multivariate logistic regression, *a priori* power analysis (G*Power 3.1; *α* = 0.05, 1-*β* = 0.80, medium effect size *f* ^2^ = 0.15, predictors = 8) indicated a minimum required sample of *N* = 109 per comparison group; the present sample far exceeds this requirement. For the Network Comparison Test (NCT), simulation evidence indicates that the observed subgroup sizes (*N* = 145, 740, 240) are sufficient to detect moderate structural differences (|Δedge| ≥ 0.20) with power >0.80 (Van Borkulo et al., 2023).

### 2.2 Data collection procedure

Data collection took place between June and December 2025 via an online survey platform, Questionnaire Star (Wenjuanxing). The research team, with the assistance of nursing education managers at each participating hospital, facilitated the distribution of the questionnaire and followed standardised procedures to ensure consistency across all data collection points. Teaching administrators within the participating hospitals sent the survey link and participation instructions to eligible nursing students on clinical placement via existing WeChat or QQ groups for nursing students. Before completing the questionnaire, all participants received a comprehensive explanation of the study’s purpose, the survey process, and their rights; informed consent was obtained electronically. The principle of voluntary participation was emphasised throughout the study to ensure the authenticity of responses. All data were collected anonymously, with no personally identifiable information recorded; data were used solely for this study, and participants’ personal privacy was strictly protected. The platform employed a mandatory completion setting, requiring all questions to be fully answered before submission, thereby eliminating missing data at the item level at source. Attention verification questions were embedded within the survey, and questionnaires failing to meet quality standards were systematically screened out in accordance with pre-survey criteria (see Section 2.1 for exclusion criteria). Technically, a mechanism to prevent duplicate submissions ensured that each participant could submit only one valid questionnaire, whilst the anonymised design further reduced the likelihood of repeated participation.

### 2.3 Research tools

#### 2.3.1 General information questionnaire

Based on a review of the literature, the researchers designed the questionnaire themselves, which included questions on age, gender, educational background, duration of clinical placement, whether the respondent was an only child, reasons for choosing the nursing programme, household income, and confidence in their ability to cope with future nursing work.

#### 2.3.2 College student environmental sensitivity scale, CSESS

The measurement was conducted using the College Students’ Environmental Sensitivity Scale (CSESS), developed by Shan et al. (2025). In 2025, for Chinese university students. The scale comprises four dimensions: Deep Perception, Deep Processing of Information, Prone to Overstimulation, and Highly Emotional, and consists of 18 items. The total score ranges from 18 to 90 points, with higher scores indicating greater environmental sensitivity. The Cronbach’s *α* coefficient for this scale is 0.86. To verify the psychometric properties of the CSESS in the sample used in this study, we conducted a confirmatory factor analysis (*CFA*) of the four-factor structure. Model fit was acceptable: *χ*^2^(133) = 314.39 (*p* < 0.001), *CFI* = 0.989, *TLI =* 0.987, *RMSEA* = 0.035 (90% *CI*: 0.028–0.046), *SRMR* = 0.082 [marginally exceeding the conventional≤0.08 threshold but within acceptable range for models of this complexity, and unlikely to substantially affect the substantive conclusions (Hu and Bentler, 1998)]. All standardised factor loadings were significant (*p<*0.001; range: 0.684–0.914). Composite reliability (*CR*>0.70) and average variance extracted (*AVE>*0.50) were satisfactory for all four subscales, supporting convergent validity; discriminant validity was also confirmed via AVE-based criteria. Sequential multi-group *CFA* further demonstrated metric invariance across sex (Δ*CFI* = 0.004, Δ*RMSEA* = 0.007, both below conventional thresholds), indicating that scale scores carry equivalent meaning for male and female participants.

#### 2.3.3 College students’ perception of failure scale

The measurement of attitudes towards failure utilised the “College Students” Attitudes Towards Failure Scale’, developed in 2024 by Chinese researchers, Niu et al. (2024). This scale comprises two relatively independent dimensions: positive and negative attitudes towards failure, and consists of 10 items. The Cronbach’s *α* coefficient for this scale is 0.83.

#### 2.3.4 Eysenck personality questionnaire short form (Chinese version)

This scale was revised by Qian et al. (2000). In 2000, the Aisenk Personality Questionnaire Short Form demonstrated good cross-cultural applicability and psychometric properties. The scale comprises four subscales, each containing 12 items, designed to measure personality traits; the Cronbach’s *α* coefficients for these subscales are 0.77, 0.78, 0.67, and 0.74, respectively.

#### 2.3.5 Career identity scale

The scale was developed by Chen et al. (2007) in 2007. It comprises five dimensions and 17 items, with a scoring range of 1 to 5 points and a total score range of 17 to 85 points; a higher score indicates a stronger career intention. The scale has a Cronbach’s *α* coefficient of 0.827.

#### 2.3.6 Self-efficacy scale

This scale was adapted into Chinese and revised by Wang et al. (2001) in 2001. It primarily measures an individual’s confidence in their ability to solve problems when faced with setbacks or difficulties. It comprises 10 items and has a total score of 40; a higher score indicates a stronger sense of self-efficacy. The scale demonstrates good reliability and validity, with a Cronbach’s *α* coefficient of 0.87.

### 2.4 Statistical methods

Statistical analysis was performed with SPSS 25.0, and network analysis models were constructed with JASP 0.96. Continuous variables following a normal distribution are expressed as (*x_ ± s*), whilst those not following a normal distribution are expressed as M (*P*_25_, *P*_75_); Categorical variables are presented as frequencies and percentages; for comparisons between groups, the chi-squared test is used for categorical variables, one-way analysis of variance (*ANOVA*) for normally distributed continuous variables, and the *Kruskal-Wallis H-test* for non-normally distributed continuous variables; common method bias (CMB) was assessed using the Harman one-way test (with a variance threshold of <40% for the first factor indicating no severe CMB), supplemented by a confirmatory factor analysis (CFA)-based unmeasured latent common method factor (ULCM) approach to ensure a more rigorous evaluation. LPA was performed using Mplus (Version 9) software. The model fit indices included: (1) the Akaike Information Criterion (*AIC*), the Bayesian Information Criterion (*BIC*) and the sample-corrected *BIC* (*aBIC*); lower values indicate better model fit (Nylund et al., 2007); (2) The closer the entropy index is to 1, the higher the classification accuracy (Celeux and Soromenho, 1996); (3) The Lo–Mendell–Rubin likelihood ratio test (*LMR*) and the bootstrap-based likelihood ratio test (*BLRT*) were used to compare adjacent models; *p* < 0.05 indicates that the Kth model fits significantly better than the (K-1) th model (Nylund et al., 2007; Lo et al., 2001). Given the exploratory nature of this study and the absence of established directional models linking environmental sensitivity to failure mindset, EBIC-GLASSO regularised partial correlation network analysis was employed rather than SEM (which requires *a priori* path specification) or simple bivariate approaches (which cannot partial out third-variable confounds), as it estimates unique conditional associations between each pair of nodes while controlling for all remaining variables and enables formal cross-profile structural comparison via the Network Comparison Test (NCT) (Van Borkulo et al., 2023; Benjamini and Hochberg, 1995). Network analysis was performed using JASP software (Version 0.96.0), with the EBIC-GLASSO (Extended Bayesian Information Criterion Graphical Least Absolute Shrinkage and Selection Operator) algorithm employed to regularise the network structure. The tuning parameter *γ* was set to 0.5, and edge weights were calculated as the normalised partial correlation coefficients between nodes after controlling for all other variables. The accuracy and stability of the network were verified using the non-parametric bootstrap method (with 1,000 samples). Networks were formally compared using the NCT with 10,000 permutations, with edge-level differences assessed after Benjamini-Hochberg false discovery rate correction. The correspondences between the nodes in the network are as follows: S1 = Deep Perception; S2 = Deep Processing Information; S3 = Prone to Overstimulation; S4 = Highly Emotional; N1 = Positive Failure Mindset; N2 = Negative Failure Mindset.

## 3 Results

### 3.1 Test for common method Bias

To evaluate CMB, we employed two complementary approaches. First, Harman’s single-factor test revealed nine factors with eigenvalues greater than 1, with the first unrotated factor accounting for 26.07% of total variance, well below the 40% threshold providing initial evidence against severe CMB (Podsakoff et al., 2003). Second, given the known limitations of Harman’s test, a CFA, based ULCM approach was adopted (Williams et al., 2010). A two-factor substantive model comprising Environmental Sensitivity (ES) and Failure Mindset (FM) (Model 1) was estimated at the parcel level, followed by a model augmented with a single common method factor (CMF) orthogonal to both substantive factors (Model 2). Although adding the CMF improved model fit (Δ*CFI* = 0.117, Δ*RMSEA* = -0.029), it accounted for an average of only 13.80% of indicator variance (range: 0.91–56.95%), remaining below the recommended 25% threshold. Collectively, these findings suggest that CMB did not substantially distort the structural relationships observed in this study.

### 3.2 Results of LPA and identification of the optimal model

This study used the four dimensions of the environmental sensitivity Scale as manifest variables to construct latent class models ranging from one to five classes. The model fit indices are shown in Table 1. Based on these indices, *AIC*, *BIC*, and *aBIC* all showed a downward trend from the one-class model to the five-class model, indicating that model fit gradually improved. The *entropy* of the 3-category model was 0.851 (>0.80), and the *p* for both the *LMR* and *BLRT* tests was <0.001, indicating that the 3-category model was significantly superior to the 2-category model. Although the 4-class model showed continued improvement in *AIC* (11,144.101), *BIC* (11,259.688), and *aBIC* (11,186.634), all lower than those of the 3-class model, with a higher entropy value (0.873) and a still-significant *LMR* (*p* = 0.0013), one class in the 4-class solution contained only 2.7% of the sample, falling below the recommended minimum threshold for reliable parameter estimation and rendering it clinically uninterpretable as a substantively meaningful profile. In contrast, the 5-class model showed a notable decline in entropy (0.816) and a substantially less significant *LMR* result (*p* = 0.0238), indicating reduced classification stability. Taking into account minimum class size adequacy, classification accuracy, and the practical interpretability and clinical significance of each profile, the three-class model was ultimately deemed optimal. When the number of latent profile categories is three, the average probability of each category belonging to its latent profile ranges from 91.0 to 94.5%, exceeding the conventional standard of 0.80 and indicating a high level of classification reliability.

**Table 1:** Fit indices for latent profile models (*n* = 1,125).

Model	*AIC*	*BIC*	*aBIC*	entropy	*P*		Proportion of each Type of sample (%)
					*LMR*	*BLRT*	
1	12782.445	12822.649	12797.239				1
2	11988.627	12053.959	12012.667	0.901	<0.001	<0.001	0.138/0.862
**3**	**11279.172**	**11369.632**	**11312.459**	**0.851**	**<0.001**	**<0.001**	**0.129/0.658/0.213**
4	11144.101	11259.688	11186.634	0.873	0.0013	<0.001	0.027/0.644/0.116/0.213
5	11020.033	11160.748	11071.812	0.816	0.0238	<0.001	0.067/0.173/0.422/0.262/0.076

Bold values indicate the three-class model, which was retained as the optimal solution. AIC, Akaike Information Criterion; BIC, Bayesian Information Criterion; aBIC, sample-size-adjusted Bayesian Information Criterion; LMR, Lo–Mendell–Rubin likelihood ratio test; BLRT, bootstrap likelihood ratio test.

**Figure 1:**
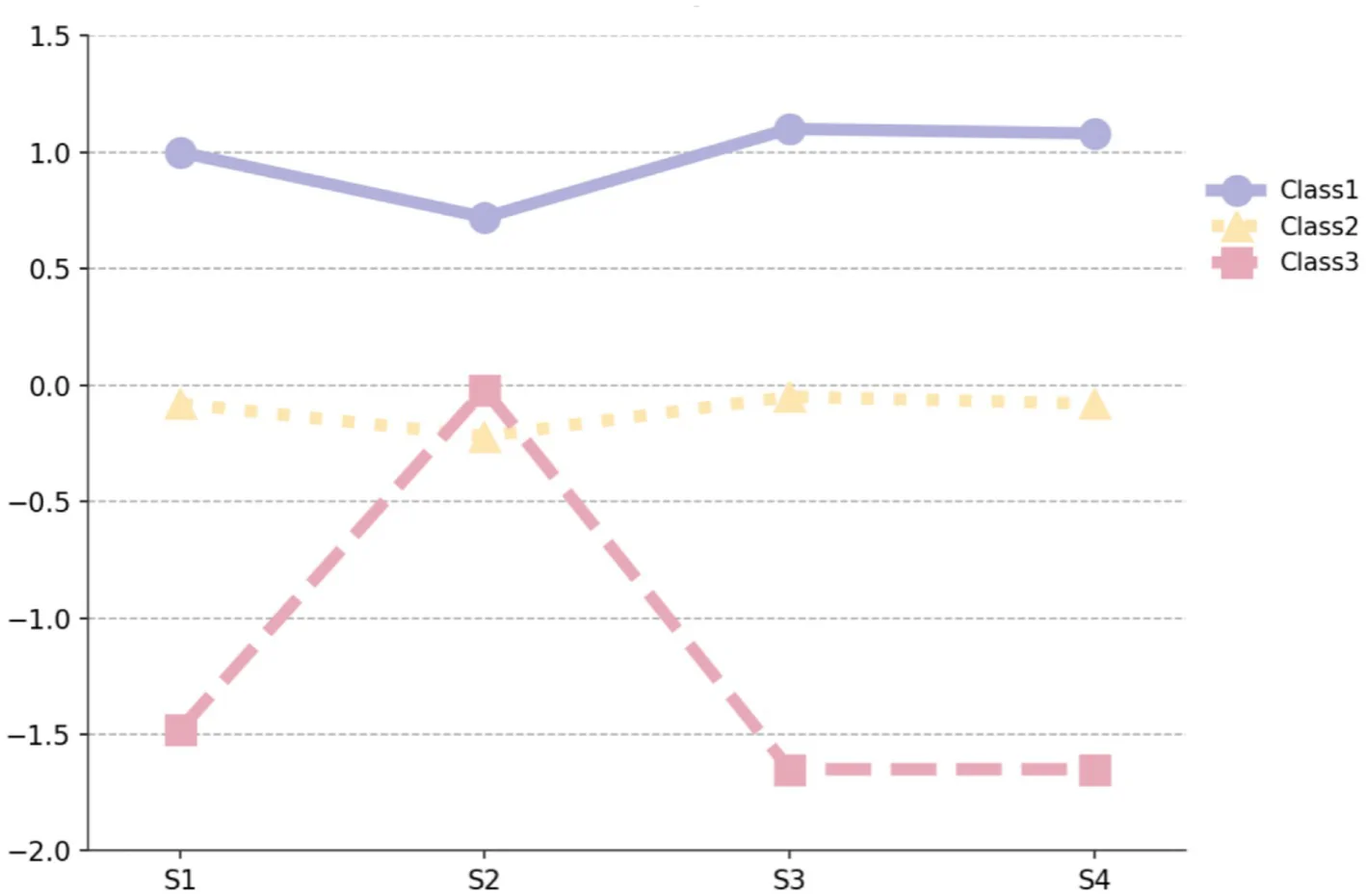
Standardised profiles of environmental sensitivity dimensions across three latent classes. S1, deep perception; S2, deep processing information; S3, prone to overstimulation; S4, highly emotional.

The three-class model categorised 1,125 nursing students into three latent categories. Figure 1 shows the standardised score profiles of each category across the four dimensions of environmental sensitivity. Based on the scoring characteristics of each category across the four dimensions, the three latent categories were named as follows: Class 1 (Highly Sensitive–Reflective): 145 individuals, accounting for 12.89%; Class 2 (Balanced-Adaptive): 740 individuals, accounting for 65.78%; Class 3 (Low-Sensitive-Rational): 240 individuals, accounting for 21.33%. These labels are descriptive shorthand derived from the observed scoring patterns across the four CSESS subscales. The terms “Reflective” and “Rational” are interpretive in nature and do not represent directly measured constructs; they should be understood as heuristic characterisations consistent with the profile configurations rather than empirically validated psychological categories.

### 3.3 Univariate analysis of potential categories of environmental sensitivity among nursing students

The results of the univariate analysis indicate statistically significant differences (*p* < 0.05) across the various categories of environmental sensitivity among nursing students, including age, gender, educational background, duration of placement, family background, motivation for choosing the profession, professional identity, and self-efficacy. No significant differences were found between the categories in terms of personality traits (introversion/extraversion) (*p* > 0.05). See Table 2 for details.

**Table 2:** Sample characteristics across three latent profiles (*n* = 1,125).

Variable	Total (*n* = 1,125)	Class1 (*n* = 145)	Class2 (*n* = 740)	Class3 (*n* = 240)	*F*/X^2^	*p*
Age (years)	20.73 ± 2.43	21.41 ± 2.48	20.76 ± 2.47	20.22 ± 2.14	11.160	<0.001
Gender						
Male	165 (14.67)	6 (4.14)	107 (14.46)	52 (21.67)	22.265	<0.001
Female	960 (85.33)	139 (95.86)	633 (85.54)	188 (78.33)		
Education level						
Secondary vocational school	220 (19.56)	19 (13.1)	145 (19.59)	56 (23.33)	24.900	<0.001
Higher vocational college	345 (30.67)	44 (30.34)	220 (29.73)	81 (33.75)		
Bachelor’s degree	425 (37.78)	49 (33.79)	293 (39.59)	83 (34.58)		
Postgraduate and above	135 (12.0)	33 (22.76)	82 (11.08)	20 (8.33)		
Internship duration (months)						
≤3	925 (82.22)	114 (78.62)	598 (80.8)	213 (88.75)	27.939	<0.001
4 ~ 6	70 (6.22)	5 (3.44)	52 (7.02)	13 (5.41)		
7 ~ 12	75 (6.67)	10 (6.89)	51 (6.89)	14 (5.83)		
>12	55 (4.89)	16 (11.03)	39 (5.27)	0 (0.0)		
Only child						
Yes	385 (34.22)	34 (23.45)	264 (35.68)	87 (36.25)	8.610	0.014
No	740 (65.78)	111 (76.55)	476 (64.32)	153 (63.75)		
Residence						
Urban	470 (41.78)	41 (28.28)	333 (45.0)	96 (40.0)	14.338	<0.001
Rural	655 (58.22)	104 (71.72)	407 (55.0)	144 (60.0)		
Reason for choosing nursing						
Self-choice	650 (57.78)	71 (48.97)	446 (60.27)	133 (55.42)	36.556	<0.001
Advised by others	340 (30.22)	40 (27.59)	235 (31.76)	65 (27.08)		
Assigned/adjusted	135 (12.0)	34 (23.45)	59 (7.97)	42 (17.50)		
Confidence in future nursing career						
Yes	975 (86.67)	107 (73.79)	653 (88.24)	215 (89.58)	24.154	<0.001
No	150 (13.33)	38 (26.21)	87 (11.76)	25 (10.42)		
Monthly household income (CNY)						
<3,000	240 (21.33)	28 (19.31)	141 (19.05)	71 (29.58)	26.642	<0.001
3,000 ~ 5,000	360 (32.0)	65 (44.83)	238 (32.16)	57 (23.75)		
>5,000	525 (46.67)	52 (35.86)	361 (48.78)	112 (46.67)		
Personality						
Introvert	355 (31.56)	54 (37.27)	235 (31.76)	66 (27.50)	4.309	0.366
Ambivert	345 (30.67)	43 (29.66)	226 (30.54)	76 (31.67)		
Extrovert	425 (37.78)	48 (33.10)	279 (37.70)	98 (40.83)		
Occupational identity	59 (52,66)	56 (51,62)	59 (51,66)	62 (54,68)	23.812	<0.001
Self-efficacy	3.0 (2.7, 3.0)	2.9 (2.60,3)	3 (2.60,3)	3 (2.8,3)	20.779	<0.001

Class1 = Highly Sensitive-Reflective Type; Class2 = Balance-Adaptive Type; Class3 = Low Sensitivity-Rational Type.

### 3.4 Analysis of differences in environmental sensitivity and failure mindset among nursing students from different potential categories

ANOVA was employed to examine differences among the three latent classes in environmental sensitivity and its sub-dimensions, as well as in the various dimensions of a failure mindset, for details, see Table 3. The statistical analysis indicated significant differences among the three classes across all measured indicators (*p* < 0.001). Specifically, regarding environmental sensitivity, Class 1’s total score (78.84 ± 16.46) and scores across all four sub-dimensions (S1-S4) were significantly higher than those of Class 2 and Class 3 (*F* = 86.89, *p* < 0.001), demonstrating the highest environmental sensitivity scores; Class 2 scored in the middle, whilst Class 3 scored the lowest. Regarding the failure mindset, Class 3 scored highest on the positive failure mindset dimension (24.81 ± 4.12), significantly outperforming Class 1 and Class 2 (*F* = 90.08, *p* < 0.001). In contrast, on the negative failure mindset dimension, Class 1 scored significantly higher than the other two groups (19.03 ± 5.61, *F* = 8.873, *p* < 0.001). Effect sizes for all between-profile comparisons are presented in Table 3 (*η*^2^ range: 0.016–0.145). Most primary outcomes yielded medium-to-large effect sizes—environmental sensitivity total score (*η^2^* = 0.134), S4 emotional reactivity (*η^2^* = 0.145), and positive failure mindset (*η^2^* = 0.138), confirming that the profile differences reflect clinically meaningful distinctions and are not merely statistical artefacts of the large sample size. The between-profile difference in negative failure mindset, though statistically significant, yielded a small effect size (*η^2^* = 0.016), falling below the conventional threshold for a medium effect (*η^2^* = 0.06); the practical magnitude of this difference should therefore be interpreted with caution.

**Table 3:** Comparison of key variables across three latent profiles.

Variable	Class1 (*n* = 145)	Class2 (*n* = 740)	Class3 (*n* = 240)	*F*	*p*	*η^2^*
Environmental sensitivity (total)	78.84 ± 16.46	62.69 ± 16.67^*^	55.73 ± 17.39^*△^	86.891	<0.001	0.134
S1	13.43 ± 2.82	12.18 ± 3.24^*^	10.96 ± 3.83^*△^	25.893	<0.001	0.044
S2	14.98 ± 3.02	12.37 ± 2.73^*^	12.59 ± 2.43^*△^	56.838	<0.001	0.092
S3	15.75 ± 5.1	13.78 ± 4.59^*^	11.41 ± 5.19^*△^	40.073	<0.001	0.067
S4	34.68 ± 9.19	24.36 ± 9.82^*^	20.76 ± 9.92^*△^	95.460	<0.001	0.145
Failure mindset (positive)	18.28 ± 5.08	22.26 ± 4.69^*^	24.81 ± 4.12^*△^	90.081	<0.001	0.138
Failure mindset (negative)	19.03 ± 5.61	17.52 ± 5.13^*^	16.69 ± 5.52^*△^	8.873	<0.001	0.016

S1, deep perception; S2, deep processing information; S3, prone to overstimulation; S4, highly emotional. ^*^p < 0.05 vs Class1; ^△^*p* < 0.05 vs Class2.

### 3.5 Multivariate logistic regression of potential categories of environmental sensitivity among nursing students

Using the different potential categories of environmental sensitivity among nursing students on clinical placement as the dependent variable (with Class 2 as the reference group), and the variables showing statistical significance in the univariate analysis as independent variables, a multivariate logistic regression analysis was performed; the results are shown in Table 4. A likelihood ratio test was conducted on the multivariate logistic regression model, yielding a *p* < 0.001, indicating a good model fit. The results showed that, compared with Class 2, being male (*OR* = 0.255, 95% *CI*: 0.106–0.617), holding a bachelor’s degree (*OR* = 0.302, 95%*CI*:0.138 ~ 0.664), being from an urban background (*OR* = 0.556, 95%*CI*: 0.357 ~ 0.864), voluntary choice of nursing as a major (*OR* = 0.347, 95%*CI*: 0.194 ~ 0.624), choice of nursing influenced by others (*OR* = 0.379, 95%*CI*: 0.207 ~ 0.691), believing they are capable of handling future nursing work (*OR* = 0.456, 95% *CI*: 0.27 ~ 0.772) were protective factors for nursing students belonging to Class 1; professional identity (*OR* = 1.041, 95%*CI*: 1.014 ~ 1.069), a monthly household income of 3,000–5,000 yuan per capita (*OR* = 1.785, 95%*CI*: 1.167 ~ 2.729) were risk factors; voluntarily choosing the nursing programme (*OR* = 0.326, 95%*CI*: 0.192 ~ 0.554) and choosing the nursing programme under the influence of others (*OR* = 0.370, 95%*CI*: 0.217 ~ 0.632) were protective factors for nursing students belonging to Class 3; general self-efficacy (*OR* = 1.876, 95%*CI*: 1.285 ~ 2.741), professional identity (*OR* = 1.022, 95%*CI*: 1.002 ~ 1.045) and a monthly per capita household income of <3,000 yuan (*OR* = 1.502, 95%*CI*: 1.024 ~ 2.202) were risk factors.

**Table 4:** Multivariate logistic regression analysis of factors associated with latent profile membership.

Comparison Group	Variable	*β*	*SE*	*Wald*	*p*	*OR*	95% *CI*
Class1 vs. Class2	Occupational Identity	0.04	0.013	8.927	0.003	1.041	(1.014 ~ 1.069)
	Male	−1.365	0.45	9.214	0.002	0.255	(0.106 ~ 0.617)
	Bachelor’s degree	−1.197	0.401	8.889	0.003	0.302	(0.138 ~ 0.664)
	Urban	−0.588	0.225	6.804	0.009	0.556	(0.357 ~ 0.864)
	Voluntarily chose nursing	−1.057	0.299	12.527	<0.001	0.347	(0.194 ~ 0.624)
	Chose nursing due to others’ influence	−0.971	0.307	10.009	0.002	0.379	(0.207 ~ 0.691)
	Competent for future nursing work	−0.784	0.268	8.564	0.003	0.456	(0.270 ~ 0.772)
	Monthly per capita family income 3,000 ~ 5,000	0.579	0.217	7.148	0.008	1.785	(1.167 ~ 2.729)
Class3 vs. Class2	Self-efficacy	0.629	0.193	10.604	0.001	1.876	(1.285 ~ 2.741)
	Occupational identity	0.022	0.011	3.747	0.047	1.022	(1.002 ~ 1.045)
	Voluntarily chose nursing	−1.12	0.27	17.234	<0.001	0.326	(0.192 ~ 0.554)
	Chose nursing due to others’ influence	−0.993	0.273	13.249	<0.001	0.37	(0.217 ~ 0.632)
	Monthly per capita family income <3,000	0.407	0.195	4.335	0.037	1.502	(1.024 ~ 2.202)

### 3.6 Comparative network analysis of potential categories of environmental sensitivity among nursing students

**Figure 2:**
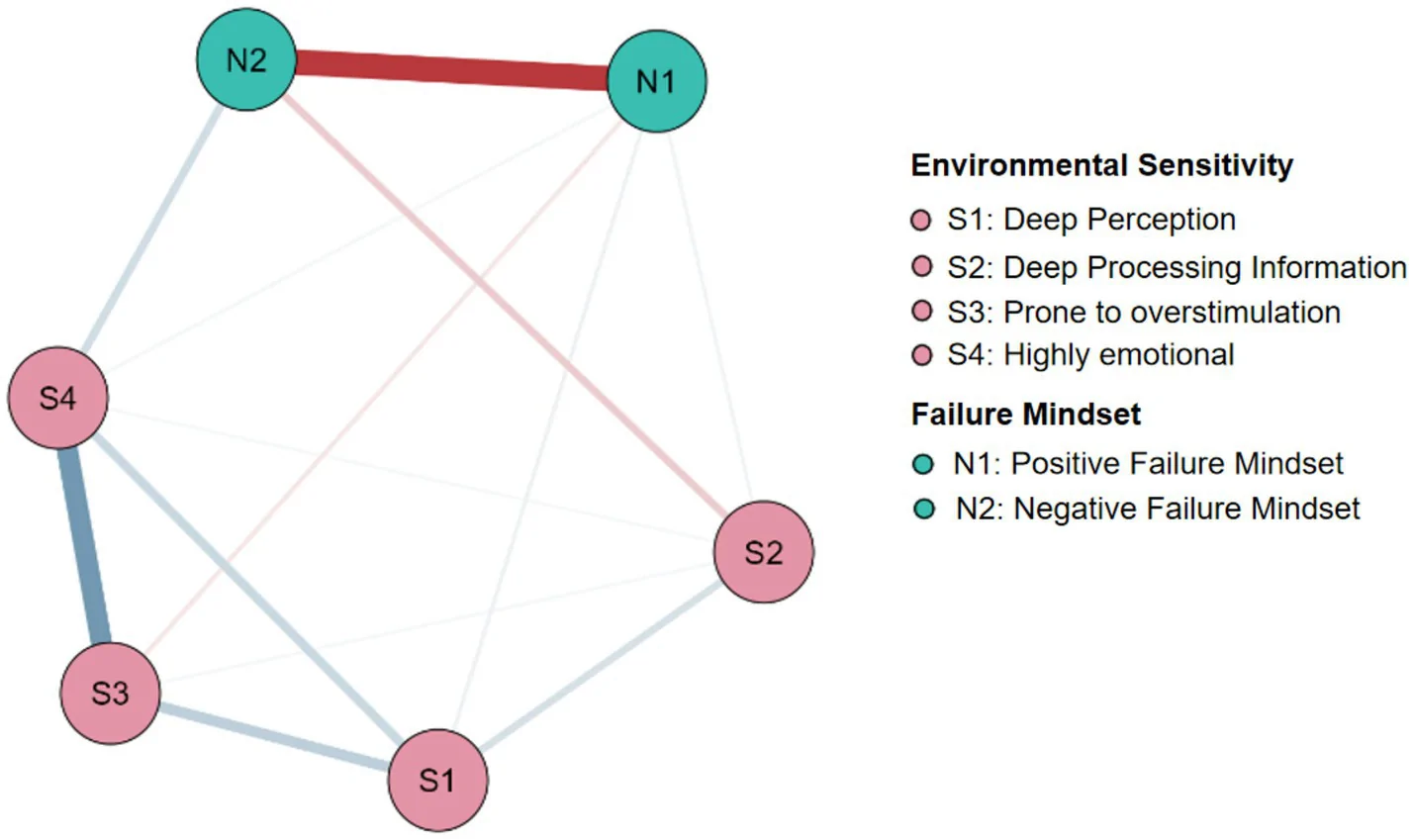
Network structure of Class 1 (Highly Sensitive-Reflective type, *n* = 145).

**Figure 3:**
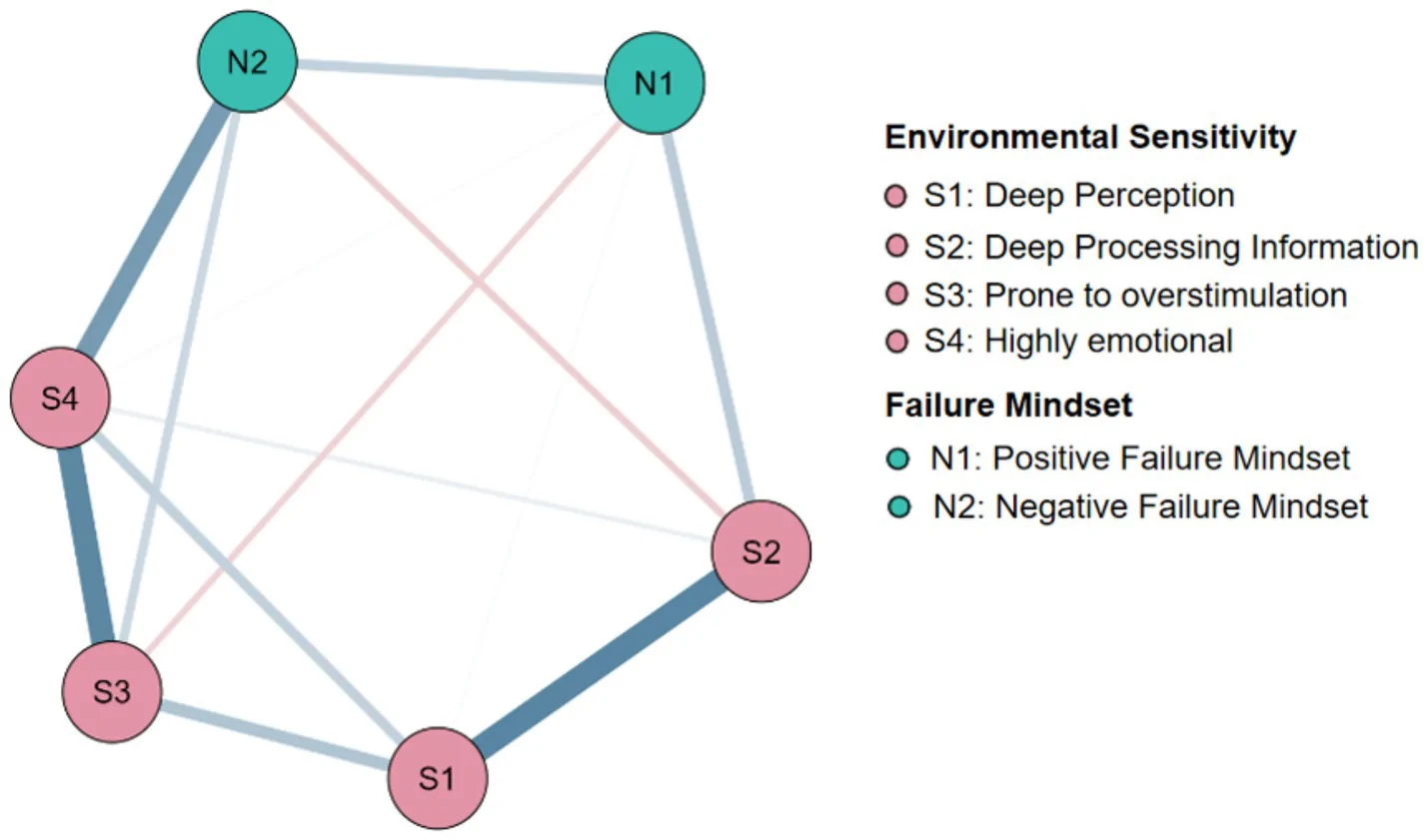
Network structure of Class 2 (Balanced-Adaptive type, *n* = 740).

**Figure 4:**
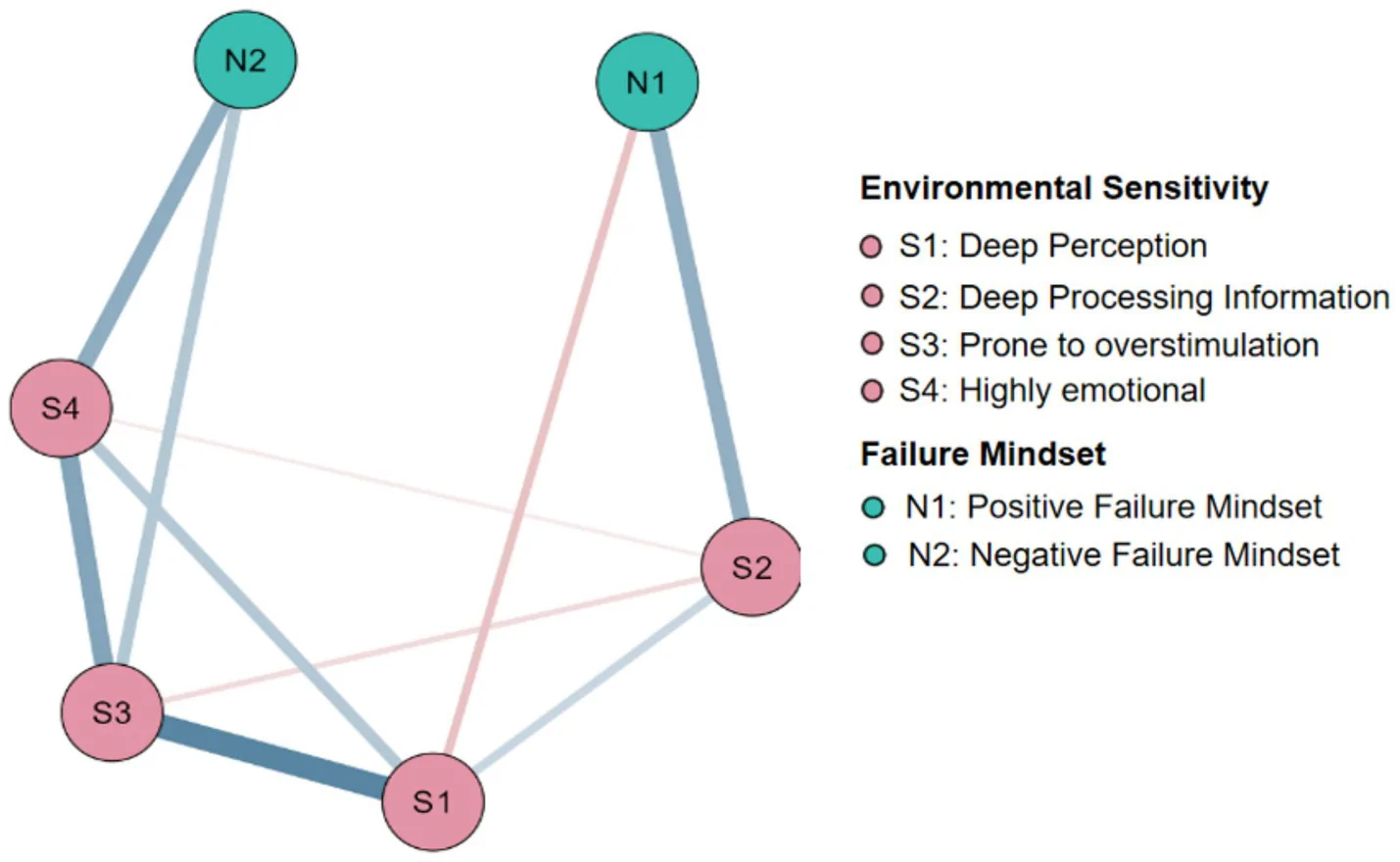
Network structure of Class 3 (Low Sensitive-Rational type, *n* = 240).

Network structures were constructed for environmental sensitivity and a failure mindset, each with three latent categories. Each network structure comprises six nodes; Class 1 and Class 2 exhibit 13 non-zero edges, whilst Class 3 exhibits 10 non-zero edges (see Figures 2–4 for details). Network analysis results indicate that the most significant feature in Class 1 is the extremely strong negative connection between N1 and N2 (*r =* −0.752), which is the strongest edge across all networks; simultaneously, S3 and S4 exhibit a strong positive correlation (*weight* = 0.633). This suggests that the highly sensitive group experiences intense cognitive conflict when faced with failure, and that external stimuli are easily transformed into strong emotional fluctuations. In the network connections of Class 2, the strongest positive connection is between S1 and S2 (weight = 0.479), indicating that this group can effectively transform perceived environmental information into deep cognitive processing. In the network connections of Class 3, the connection between N1 and N2 disappears, indicating that positive and negative attitudes towards failure are relatively independent in the low-sensitivity group; the strongest connection appears between S1 and S3 (*weight* = 0.508). To formally evaluate whether these structural differences were statistically significant, a Network Comparison Test (NCT) was conducted using permutation-based methods (10,000 permutations; see Supplementary Tables S3,S4). All three pairwise comparisons yielded significant M-statistics: Profile 1 vs. Profile 2 (*M* = 0.951, *p*<0.001), Profile 1 vs. Profile 3 (*M* = 0.749, *p<*0.001), and Profile 2 vs. Profile 3 (*M =* 0.279, *p* = 0.011), confirming that the three networks are structurally non-equivalent. At the edge-specific level, after Benjamini-Hochberg (*BH*) false discovery rate correction, the N1-N2 edge was the most discriminating feature across comparisons (P1 vs. P2: |Δ*w*| = 0.951; P1 vs. P3: |Δ*w*| = 0.749; both P(*BH*)<0.001), statistically confirming that the strong negative coupling between positive and negative failure mindsets is a structural signature unique to the highly sensitive profile. In the Profile 1 vs. Profile 2 comparison, the S1-S2 edge also survived *BH* correction (|Δ*w*| = 0.305, *P*(*BH*) = 0.015). In the Profile 1 vs. Profile 3 comparison, five additional edges survived correction: S3-N2 (|Δ*w*| = 0.329, *P*(*BH*)<0.001), S1-N1 (|Δ*w*| = 0.299, *P*(*BH*)<0.001), S3-S4 (|Δ*w*| = 0.282, *P*(*BH*) = 0.022), S3-N1 (|Δ*w*| = 0.254, *P*(*BH*) = 0.024), and S1-S3 (|Δ*w*| = 0.239, *P*(*BH*) = 0.032), further corroborating the disproportionate strengthening of the “stimulus-emotion amplification” pathway in Profile 1. In the Profile 2 vs. Profile 3 comparison, four edges survived correction: S1-S3 (|Δ*w*| = 0.279, *P*(*BH*)<0.001), S1-S2 (|Δ*w*| = 0.272, *P*(*BH*)<0.001), S3-N1 (|Δ*w*| = 0.239, *P*(*BH*) <0.001), and S1-N1 (|Δ*w*| = 0.246, *P*(*BH*) = 0.007).

**Figure 5:**
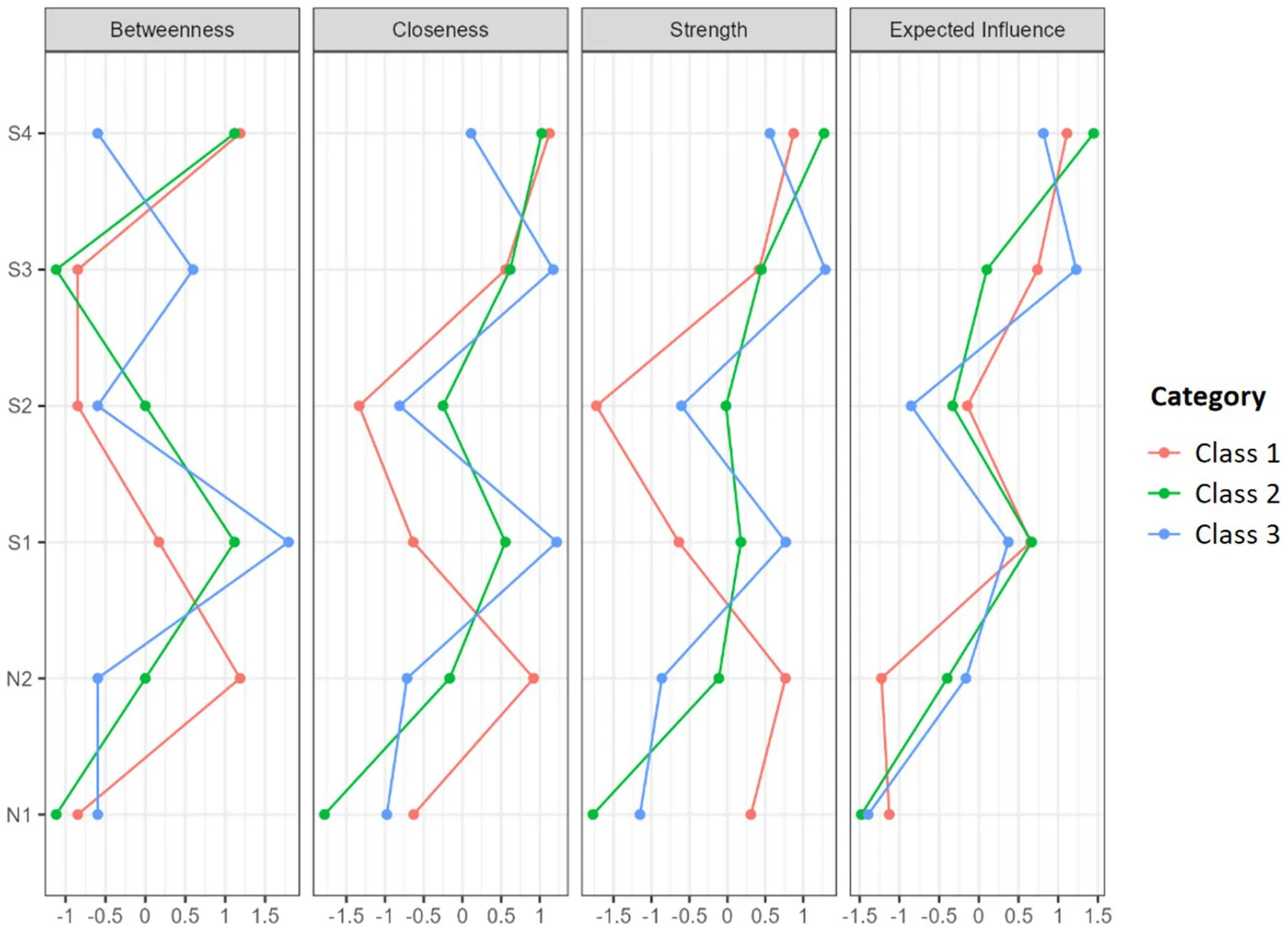
Node centrality indices across three network structures. Class 1: Highly sensitive-reflective type; Class 2: Balanced-adaptive type; Class 3: Low sensitivity-rational type.

Regarding node centrality, this study comprehensively examined four indicators: betweenness centrality, closeness centrality, strength centrality, and expected influence (EI). For Class 1, node S4 showed high centrality in EI, betweenness centrality, and strength centrality, and was a key driver of psychological network symptoms in this subgroup; whereas N2 and N1 exhibited significant negative expected influence. In Class 2, S4 scored significantly higher than other nodes across all centrality metrics, followed by S1. Unlike the first two groups, in Class 3, S3 was the most central node, followed by S4. For further details, see Figure 5.

**Figure 6:**
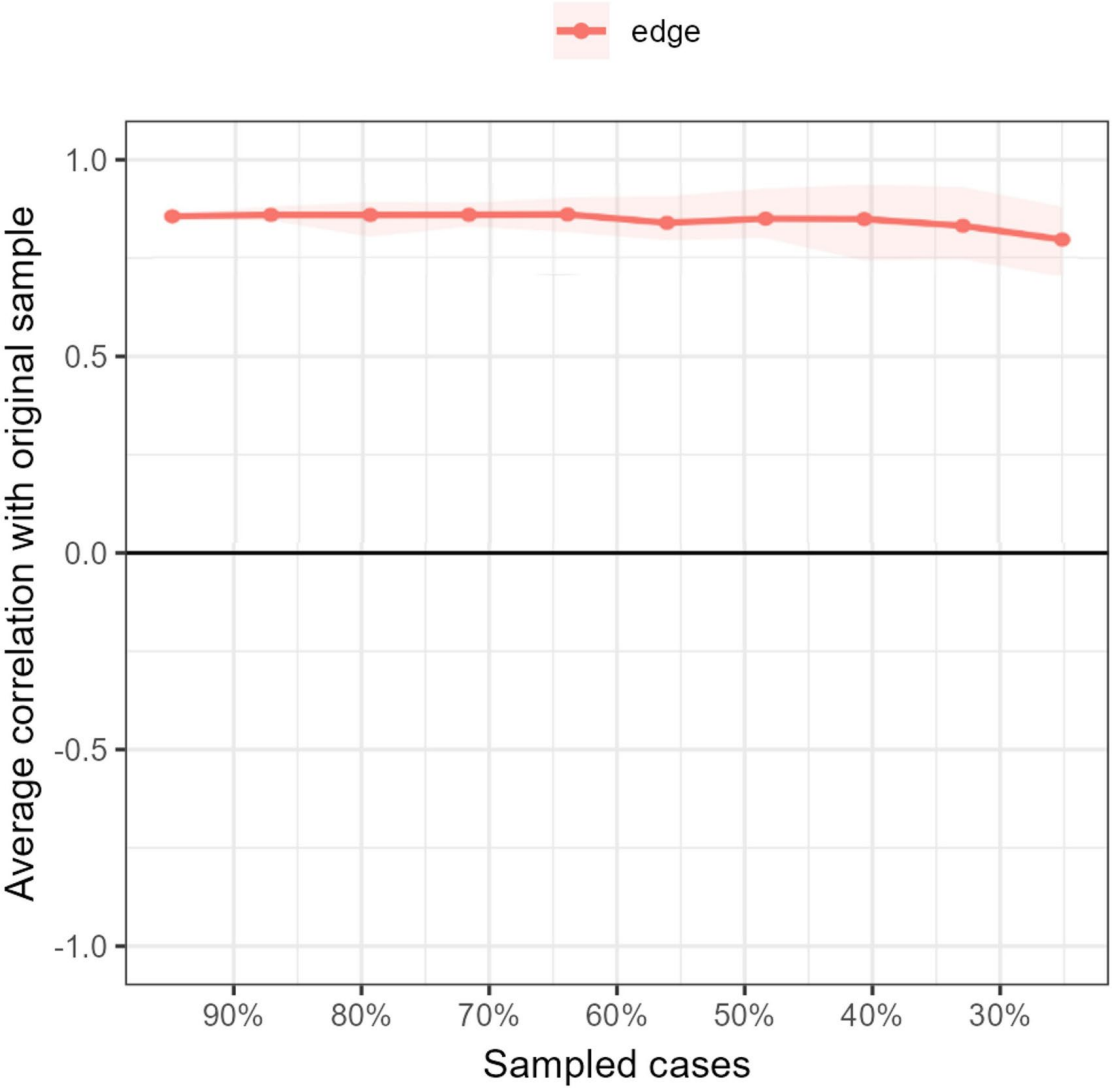
Edge weight accuracy for the Class 1 network.

**Figure 7:**
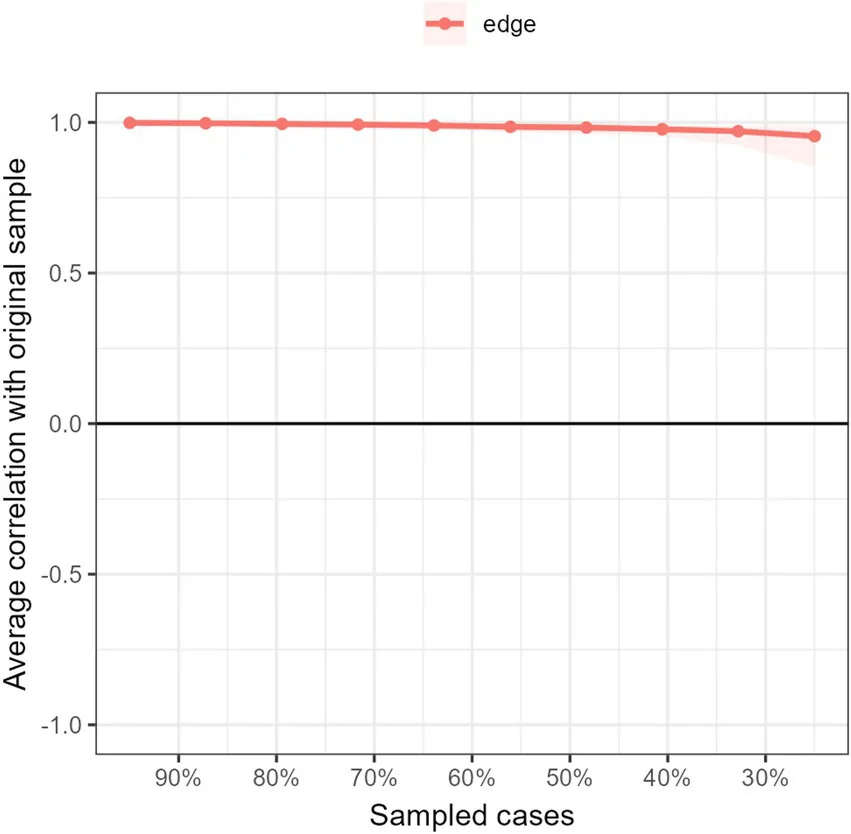
Edge weight accuracy for the Class 2 network.

**Figure 8:**
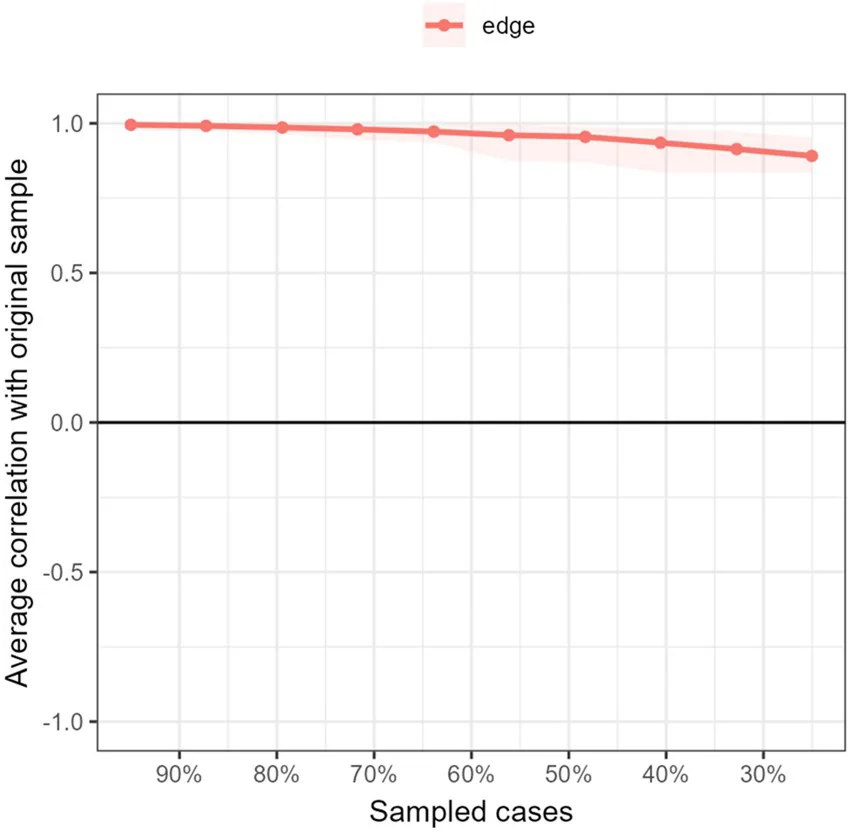
Edge weight accuracy for the Class 3 network.

**Figure 9:**
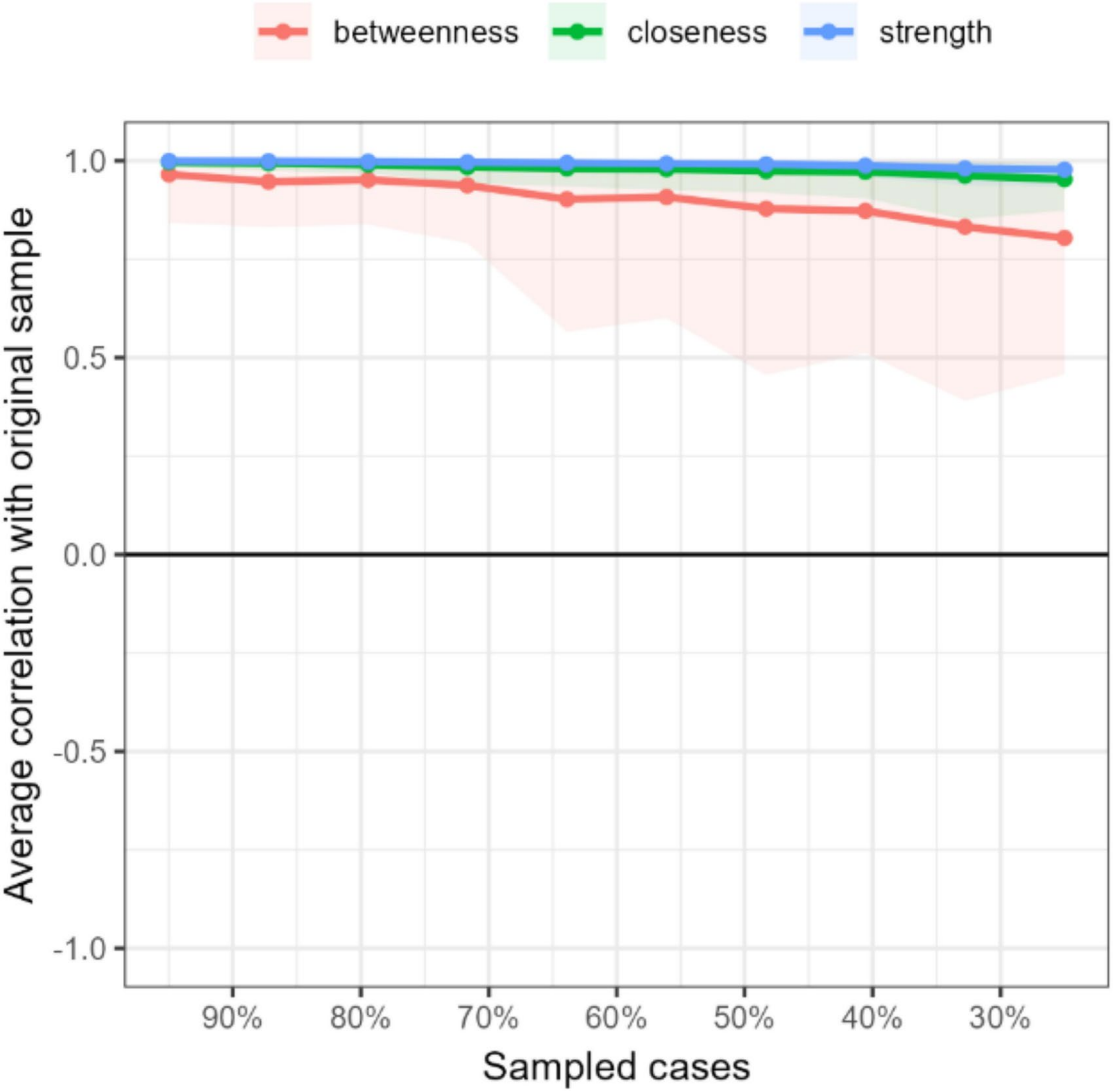
Node centrality stability for the Class 1 network.

**Figure 10:**
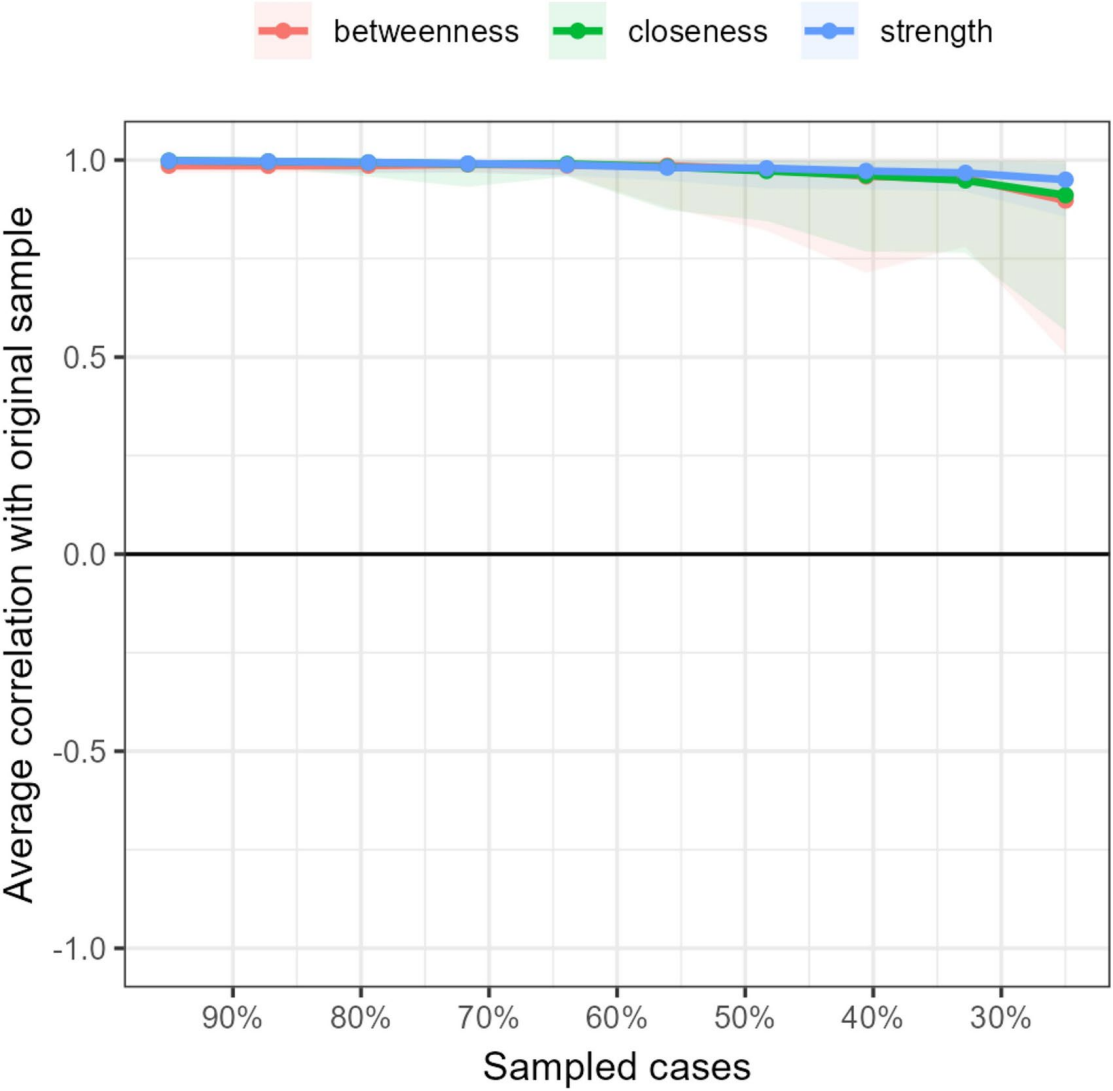
Node centrality stability for the Class 2 network.

**Figure 11:**
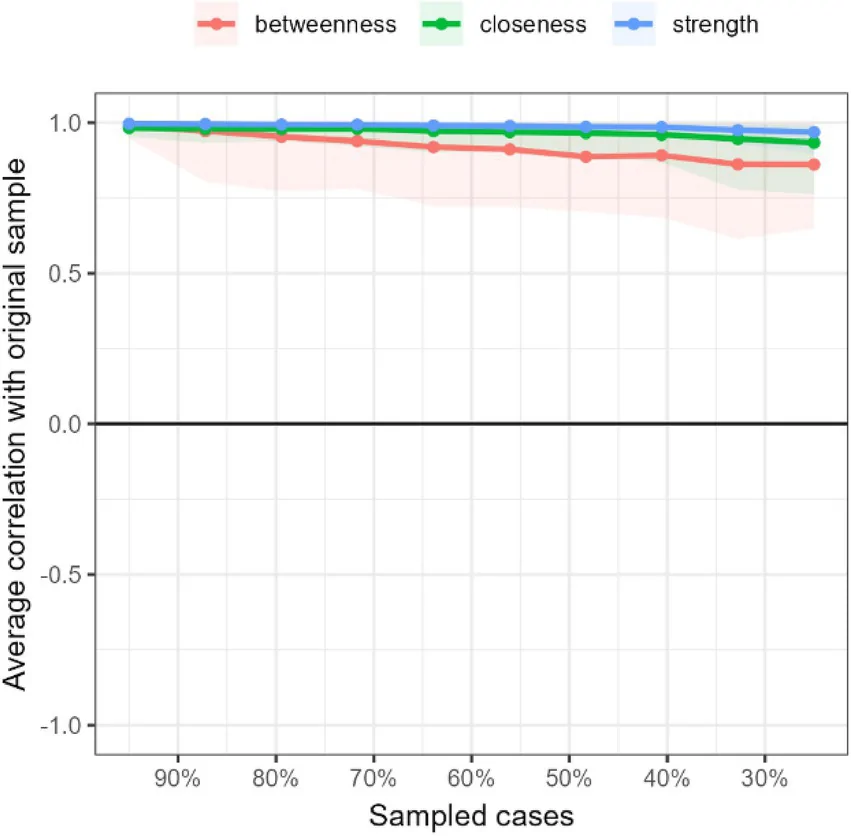
Node centrality stability for the Class 3 network.

To verify the reliability of the above results, this study employed a non-parametric bootstrap method (with 1,000 samples) to test the accuracy and stability of the networks. The results of the edge accuracy tests (Figures 6–8) show that the 95% confidence intervals for each edge across the three network categories are narrow and do not cross zero, indicating that the estimated edge weights are accurate and reliable. The results of the node centrality stability tests (Figures 9–11) revealed differential stability patterns across centrality indices and subgroups. Strength centrality demonstrated excellent stability across all three classes, with CS-coefficients at *r* = 0.7 of 0.82, 0.85, and 0.79 for Class 1, Class 2, and Class 3, respectively, all substantially exceeding the recommended threshold of 0.50, indicating that strength centrality estimates remained highly consistent even after the random removal of 75% of participants. Closeness centrality exhibited good stability in Class 2 and Class 3 (CS = 0.67 and 0.61, respectively); although the CS value for Class 1 (CS = 0.44) fell marginally below the 0.50 threshold, this was considered acceptable given the relatively smaller subsample size. For betweenness centrality, adequate stability was observed in Class 2 and Class 3 (CS = 0.54 and 0.58, respectively); however, the markedly lower CS value in Class 1 (CS = 0.21), together with the substantially widened confidence intervals observed in Figure 9, suggests that betweenness centrality estimates for this subgroup should be interpreted with caution. Taking these test results into account, the network analysis findings of this study demonstrate good accuracy and stability, and the conclusions are reliable. For precise centrality values and edge weights, see Supplementary Tables S1,S2. Specifically, the CS-coefficients for strength centrality were CS(r = 0.7) = 0.82, 0.85, and 0.79 for Class 1, Class 2, and Class 3, respectively, all exceeding the recommended threshold of 0.50 and indicating excellent stability. For closeness centrality, CS-coefficients were 0.44, 0.67, and 0.61 for Class 1, Class 2, and Class 3, respectively; the value for Class 2 and Class 3 indicated good stability, whilst that for Class 1, although below 0.50, remained within an acceptable range given the smaller subsample size (*n* = 145). For betweenness centrality, CS-coefficients were 0.21, 0.54, and 0.58 for Class 1, Class 2, and Class 3, respectively; the substantially lower value for Class 1 (CS = 0.21) warrants caution in interpreting betweenness centrality estimates for this subgroup, consistent with the widened confidence intervals observed in Figure 9.

Importantly, global network strength did not differ significantly across any pairwise NCT comparison (Profile 1 vs. Profile 2: *S* = 0.302, *p* = 0.348; Profile 1 vs. Profile 3: *S* = 0.342, *p* = 0.232; Profile 2 vs. Profile 3: *S* = 0.041, *p* = 0.895; global network strength: *P*1 = 3.194, *P*2 = 2.892, *P*3 = 2.851), indicating that whilst the topological configurations differ substantially, overall connectivity levels are broadly preserved across profiles. This dissociation between structural topology and global strength suggests that environmental sensitivity profiles differ not in how strongly individuals are psychologically activated overall, but in how their psychological resources are organised and routed-providing formal statistical grounding for the profile-specific intervention targets identified in this study.

## 4 Discussion

This study represents the first attempt to identify heterogeneous subgroups of environmental sensitivity among nursing students on clinical placement using LPA and to examine their associations with failure mindsets. Furthermore, network analysis was employed to explore the differential interrelationships between environmental sensitivity dimensions and failure mindset components across subgroups. The findings revealed three distinct profiles of environmental sensitivity: Highly Sensitive-Reflective type (Class 1, 12.89%), Balanced-Adaptive type (Class 2, 65.78%), and Low Sensitivity-Rational type (Class 3, 21.33%). These subgroups exhibited significant differences in failure mindsets, with Class 1 showing the most negative and Class 3 the most positive; inter-profile differences for the primary outcomes were underpinned by medium-to-large effect sizes (*η^2^* = 0.134–0.145 for the main comparisons), attesting to their practical clinical relevance. Network analysis further elucidated distinct connectivity patterns across subgroups, highlighting the centrality of emotional reactivity in Class 1 and the relative independence of positive and negative failure mindsets in Class 3. These findings extend the current understanding of environmental sensitivity in nursing education and offer actionable insights for tailored interventions.

### 4.1 Potential demographic characteristics of nursing students’ environmental sensitivity

This study used LPA to identify three subgroups of environmental sensitivity among nursing students: the Highly Sensitive-Reflective type (Class 1), the Balanced-Adaptive type (Class 2), and the Low Sensitive-Rational type (Class 3). The identification of these three subgroups aligns closely with the theoretical framework of environmental sensitivity, supporting the core assertion in Aron et al.’s (Aron and Aron, 1997) theory of Highly Sensitive Persons (HSP) regarding fundamental differences in the depth of perception between individuals. Furthermore, it provides further evidence for the applicability of Belsky et al.’s “Differential Susceptibility Theory” within the cohort of nursing students (Belsky and Pluess, 2009). It should be noted that the profile labels, notably “Reflective” for Class 1 and “Rational” for Class 3, are theoretically informed descriptors derived from the observed CSESS subscale patterns, specifically the elevated S2 (Deep Processing of Information) scores in Class 1 and the markedly lower S3 and S4 scores in Class 3 (Aron and Aron, 1997; Lionetti et al., 2018; Liss et al., 2008), rather than directly measured psychological constructs, and should be understood as theoretically grounded characterisations rather than empirically validated categories.

Although the Highly Sensitive-Reflective Type (Class 1) represents the smallest proportion (12.89%), its scores on the two dimensions of strong emotional reactivity (S4) and deep information processing (S2) are significantly higher than those of the other two categories. This is consistent with the findings of neuroimaging studies by Acevedo et al. (2014). Although the present study did not directly assess neurobiological variables, the Class 1 profile pattern, is broadly consistent with this neuroimaging literature. Whether shared neurobiological mechanisms underlie the current self-report network findings remains an open question for future neuroimaging research. The “Reflective” label for this profile is informed by these theoretical accounts of deeper cognitive elaboration, consistent with the elevated S2 scores observed. This group is associated with a high degree of clinical insight and empathic potential, potentially enabling trainees to keenly detect subtle emotional changes in patients, which may aid in building high-quality nurse–patient relationships; however, in high-pressure situations such as operational errors or criticism from supervisors, the tendency to amplify negative stimuli observed in this profile may be associated with increased risk of emotional exhaustion, consistent with previous research findings in nursing populations showing a positive correlation between high sensitivity and occupational burnout (Golonka and Gulla, 2021).

The Balanced-Adaptive type (Class 2) was the most prevalent category in the sample, accounting for 65.78%. Scores across all dimensions were moderate, indicating a dynamic balance between environmental awareness and emotional regulation. This distribution aligns with the pattern observed in previous studies using LPA to examine the psychological traits of university students, in which the intermediate category typically accounted for the highest proportion (Aron and Aron, 1997), suggesting that this group may represent a typical cohort of nursing students in terms of clinical adaptability. In clinical practice, such trainees can promptly identify patient needs and environmental changes, whilst being less prone to emotional strain from excessive sensitivity; consequently, they have strong potential for professional adaptation.

The Low-Sensitivity-Rational Type (Class 3) accounted for 21.33% of the sample, scoring lowest on the dimensions of susceptibility to overstimulation (S3) and emotional reactivity (S4), a pattern consistent with strong emotional stability and a tendency towards rational thinking, the basis for the descriptive “Rational” label, which, as noted, is a theoretically informed inference rather than a directly measured characteristic. Trainees in this profile may be better positioned to remain relatively calm and objective in high-pressure clinical environments, which could facilitate rapid decision-making during emergencies. Consistent with the findings of Liss et al. (2008), given the relatively weak ability of low-sensitivity individuals to perceive socio-emotional cues, this group may have difficulty recognising subtle emotional signals from patients. This could pose a risk to clinical quality in patient–nurse communication scenarios that require a high degree of empathy.

### 4.2 The relationship between potential categories of environmental sensitivity and a failure mindset

This study found significant differences in failure mindset across three latent categories, with the patterns of variation closely aligning with theoretical expectations. Class 1 exhibited the highest negative failure mindset and the lowest positive failure mindset. Specifically, high environmental sensitivity, as a latent vulnerability, is associated with a tendency towards negative failure attributions in the sustained stress environment of clinical placements, potentially through the amplification of cognitive processing of negative events (Lionetti et al., 2018). When highly sensitive trainees encounter clinical setbacks such as procedural errors or criticism from supervisors, their heightened awareness of negative environmental cues may be associated with an excessive focus of attentional resources on the failure event itself, alongside a cognitive bias of “failure salience” and a negative attribution pattern of “fixed personal ability” (Memari et al., 2024). This aligns with Dweck’s theoretical mechanism of a fixed mindset, in which individuals tend to internalise failure as a deficiency in ability (Haimovitz and Dweck, 2016). If such cognitive patterns are not addressed promptly, they may adversely affect trainees’ learning motivation, skill acquisition, and even long-term career development (Borda-Mas et al., 2025). In contrast, the limited depth of processing of environmental stimuli among low-sensitivity groups is associated with maintaining a relatively objective cognitive perspective when faced with the same failure scenarios; they tend to view failure as a situational, modifiable event, thereby sustaining a positive growth mindset (Kondratowicz, 2022; Song et al., 2020).

Conversely, the low-sensitivity-rational type (Class 3) exhibits the most ideal cognitive pattern regarding failure among the three groups. From the perspective of emotion regulation theory, the limited reactivity of low-sensitivity individuals to negative environmental stimuli essentially constitutes a natural cognitive buffering mechanism (Acevedo et al., 2014; Song et al., 2020). When a failure occurs, lower levels of emotional arousal may be associated with a more stable cognitive baseline that facilitates positive reappraisal. Future research incorporating neurobiological measures (e.g., autonomic indices or neuroimaging) could help clarify the mechanisms underlying this pattern (Sun et al., 2023). This aligns closely with the mechanism of cognitive reappraisal as a pre-focal strategy within Gross’s Process Model of Emotion Regulation (Gross, 1998; Uusberg et al., 2023). However, it should be noted that Class 3’s advantage in terms of a positive mindset towards failure does not necessarily imply that their overall psychological adaptation is optimal; this group’s lower scores on negative mindset may partly stem from a natural lack of depth in their perception of failure events, rather than a genuinely positive, growth-oriented cognitive perspective (Wong et al., 2023). In other words, they may not have fully experienced the failure and, consequently, failed to extract learnable information from it (Dweck and Yeager, 2019). This represents a fundamental divergence from Carol Dweck’s definition of a growth mindset: a genuine positive mindset towards failure should be premised on a full perception of failure and active meaning-making, rather than at the expense of emotional numbness or cognitive avoidance (Sik et al., 2024). If the positive failure scores of Class 3 are primarily attributable to the latter pathway, they may face the latent risk in clinical practice of being ‘insensitive to failure and unable to learn from it’; yet this risk is precisely difficult to identify at the level of standard questionnaire scores.

The Balanced-adaptive type (Class 2) exhibited moderate levels of both positive and negative failure attitudes, maintaining a relatively balanced tension between the two. It is worth noting, however, that Class 2 exhibited lower levels of negative failure mindset than Class 1, and a statistically significant difference remained when compared with Class 3 (*F* = 8.873, *p* < 0.001); however, the effect size (*η^2^* = 0.016) suggests that the actual magnitude of this difference is relatively limited. Viewed through the lens of the Stress Accumulation Model, the psychological resilience of the balanced-adaptive group may be context-dependent; in the event of a single failure, their balanced perceptual-processing integration capacity is sufficient to maintain positive attributions of failure (Thomas and Revell, 2016); however, during the high-stress phase in the latter stages of clinical placement, where failure events frequently accumulate, the sustained depletion of cognitive resources may gradually erode this equilibrium, lowering the threshold for the activation of negative mental states (Liu et al., 2022; Cheng et al., 2023). This inference is consistent with longitudinal observations of the ‘honeymoon effect’ in previous nursing placement studies (Lavoie-Tremblay et al., 2022; Sonmez et al., 2023), in which trainees with greater adaptability often experience phasic fluctuations in their psychological state during the middle and later stages of their placement. Consequently, intervention strategies for Class 2 should not be limited to immediate psychological support. Still, they should focus on building sustainable cognitive resilience reserves to fundamentally enhance their psychological capital for coping with cumulative clinical stress. Beyond individual differences, the cultural meaning of failure in China may further shape nursing students’ failure mindset. In the Chinese context, academic and professional failure may carry strong social and familial implications, including expectations for academic achievement, stable employment, and family responsibility. For nursing interns, clinical errors or negative evaluations from supervisors may therefore be perceived not only as temporary learning setbacks but also as threats to future career development and family expectations. These cultural expectations may intensify students’ fear of failure during the transition from nursing education to clinical practice, particularly among students with higher environmental sensitivity (Zhou et al., 2025; Chen et al., 2025). Therefore, culturally sensitive educational interventions are needed to help students reframe clinical errors as part of professional development rather than as indicators of personal inadequacy or failure to meet external expectations.

### 4.3 Mechanisms of inter-category differences revealed by network analysis

It should be noted that network centrality indices, as estimated from cross-sectional data, reflect statistical connectivity patterns rather than causal influence or underlying psychological mechanisms. Recent methodological literature has cautioned against interpreting centrality as a proxy for causal priority or psychological importance (e.g., Bringmann et al., 2019; Dablander and Hinne, 2019). Accordingly, the centrality-based findings reported here should be understood as hypothesis-generating observations that warrant confirmation through longitudinal or experimental designs.

Network analysis revealed the fundamental differences in the underlying psychological mechanisms linking environmental sensitivity and a failure mindset across the three subgroups at the level of variable interrelationships. The most characteristic finding in Class 1 networks is the extremely strong negative connection between N1 and N2 (*r* = −0.752). This implies that the two form a dynamically coupled system of competitive inhibition; when a negative failure mindset is activated, it shows a strong negative association with the positive mindset node via this connection, and vice versa. This structural feature is consistent with the pattern whereby the highly sensitive group exhibits the coexistence of the highest negative failure mindset and the lowest positive failure mindset; the two mindsets do not vary independently, but rather form a dynamic relationship of “one is strong, the other is weak” through strong coupling. At the network topology level, this antagonistic structure suggests that once a negative failure mindset is activated in highly sensitive individuals, concurrent activation of a positive mindset may be structurally inhibited at the psychological level. A theoretically plausible, though speculative-neurocognitive account would attribute this to resource competition in executive control (Berboth and Morawetz, 2021). Given that the present study relied entirely on self-reported questionnaire data, no inference about specific neural substrates can be drawn. Testing this neurocognitive hypothesis would require neuroimaging or psychophysiological designs. The present network evidence is therefore best treated as hypothesis-generating (Cui et al., 2024). This is consistent with Joormann’s neurocognitive model of impaired cognitive control function in individuals susceptible to depression (Joormann and Gotlib, 2010; Gotlib and Joormann, 2010). At the level of self-reported network structure, the present data suggest that once highly sensitive trainees enter a state of negative failure attribution, disengaging from that state may be psychologically effortful rather than simply a matter of insufficient willpower. Whether this pattern reflects the structural neural constraints proposed by Joormann’s model cannot be determined from the current cross-sectional, self-report data and would require experimental or neuroimaging validation to confirm.

Furthermore, the strong positive connection between S3 and S4 in Class 1 establishes an efficient ‘stimulus-emotion amplification’ transmission pathway; external negative stimuli show strong associations with intense emotional arousal via S3, and the resulting disturbance is linked to the broader network via S4’s pivotal role. This implies that any negative stimulus from the clinical environment, be it operational errors, criticism from supervisors, or nurse–patient conflicts, may be associated with cascading amplification responses within this group’s psychological system and with the accumulation of psychological burden (Homberg and Jagiellowicz, 2022). The finding that S4 shows the highest centrality in the Class 1 network indicates a potential network-level correlate of psychological vulnerability in highly sensitive individuals, suggesting a possible focus for future intervention research, pending experimental validation. Although S4 was the most central node in the Highly Sensitive-Reflective subgroup, the strong positive connection between S3 and S4 indicates that proneness to overstimulation also plays an important role in this subgroup. This S3-S4 connection may represent a “stimulus-emotion amplification” pathway, through which sensory and cognitive overload is rapidly transformed into emotional reactivity (Costa-López et al., 2024). In clinical settings, this may manifest as difficulty coping with alarm noise, rapid task switching, crowded ward environments, patient suffering, or heightened anxiety after procedural mistakes. Through its strong connection with S4, overstimulation may trigger a cascading emotional response that exceeds the intensity of the original clinical stimulus and contributes to the accumulation of psychological burden. Therefore, interventions for highly sensitive nursing students should not only target emotional regulation but also reduce overstimulation and improve coping with sensory and emotional demands in clinical placements (Li et al., 2024).

The most striking feature of the Class 2 network is its highest connection density among the three groups (13 non-zero edges), reflecting the richest integration of interactions among variables within this group’s psychological system. Centred on the strong positive connection between S1 and S2 (*weight* = 0.479), this network structure functionally corresponds to the efficient integration of the first two stages of the cognitive cycle of “concrete experience-reflective observation-abstract conceptualization” in experiential learning theory (Cheng et al., 2025). This group not only possesses the ability to perceive subtle changes in the clinical environment but is also capable of instantly transforming this perceptual information into meaningful, deep cognitive processing, thereby establishing an efficient pathway from perceptual input to the construction of experience (Salem et al., 2025). Of even greater theoretical significance is the fact that the characteristic of Class 2, having the highest network connectivity density, itself carries important psychological functional implications. Higher connectivity density typically indicates that the psychological system possesses stronger state propagation and integration capabilities; that is, when a change occurs at a particular node, it can rapidly influence other nodes through a rich network of pathways, thereby enabling the entire system to maintain greater dynamic coordination and adaptability (Borsboom et al., 2021). This aligns closely with the behavioural manifestations of balanced-adaptive groups, who can flexibly draw on diverse cognitive resources and maintain dynamic psychological equilibrium when faced with clinical setbacks (Shen et al., 2024). Although S4 also occupies the most central position within Class 2, its edge weights are significantly lower than those in Class 1. This suggests that the influence of emotional reactivity is effectively diluted by a more balanced network structure in this group; the disruptive energy of emotional activation is dispersed across multiple parallel pathways during propagation, rather than being concentrated along a single strong edge as in Class 1. This constitutes an important network mechanism underpinning the psychological resilience of Class 2.

The formal NCT further substantiates these observations. Permutation-based comparisons (10,000 permutations) revealed significant differences in global network topology across all three profile pairs (Van Borkulo et al., 2023), confirming that the three networks represent structurally distinct configurations rather than sampling variants of a common architecture. Conversely, global network strength did not differ significantly across any comparison, indicating that the profiles are equivalent in overall connectivity density but diverge in how psychological resources are internally organised and routed. At the edge-specific level, after Benjamini-Hochberg correction (Benjamini and Hochberg, 1995), the N1-N2 edge consistently emerged as the most discriminating feature, statistically confirming that the competitive inhibition between positive and negative failure mindsets is a structural signature of the highly sensitive profile rather than a universal network property. The additional significant edges involving S3 in Profile 1 vs. Profile 3 comparisons corroborate the heightened stimulus-emotion amplification pathway identified in Class 1. Collectively, these NCT findings provide formal statistical grounding for the profile-specific intervention targets discussed above.

The most striking finding in the Class 3 network is the complete disappearance of the connection between N1 and N2; this absence in itself carries rich psychological information. Within the framework of network analysis, the reduction of an edge to zero implies that, after controlling for all other nodes, there is no significant conditional correlation between the two variables (Epskamp and Fried, 2018); that is, positive and negative failure mindsets do not constitute a coupled system of mutually competitive activation in the low-sensitivity group, but rather operate as genuinely independent entities at the level of psychological structure (Watson et al., 1988). This state indicates that low-sensitivity individuals are capable of simultaneously accommodating, within the same cognitive system, both an objective acknowledgement of the negative aspects of failure and a positive expectation of growth potential, without triggering resource competition or mutual inhibition between the two (Hu et al., 2025). The psychological origins of this state require careful analysis; its formation may stem from genuine cognitive integration driven by emotional buffering mechanisms (Gross, 2015), or it may result from perceptual blunting, whereby neither mindset is fully activated, thus presenting a superficial independence (Rottenberg et al., 2005). Network analysis alone cannot directly distinguish between these two pathways (Borsboom and Cramer, 2013); however, combining topological evidence from Class 3, where S3 serves as the network’s central node, with the threshold-dependent cognitive activation pattern revealed by the strong connections between S1 and S3, it can be inferred that the contribution of the perceptual blunting pathway to the decoupling of N1 and N2 in this group should not be underestimated. This inference carries significant implications for clinical practice: supervisors should not simply interpret high scores as indicative of a positive failure mindset and low scores as indicative of a negative mindset in Class 3 as indicative of an optimal state of mental health. Instead, they should use methods such as in-depth interviews or Ecological Momentary Assessment (EMA) to further identify the underlying mechanisms, thereby formulating intervention strategies that genuinely address their psychological needs.

### 4.4 Insights from the analysis of influencing factors

Multivariate logistic regression analysis revealed multidimensional factors influencing the classification of nursing students’ environmental sensitivity, including motivation for choosing the profession, professional identity, self-efficacy, and demographic characteristics; these factors exhibited distinct patterns in the mechanisms underlying the formation of Class 1 and Class 3. Concerning motivations for choosing the profession, compared with students allocated to the nursing programme, both voluntary choice and choosing nursing on the advice of others were protective factors for assignment to Class 1, and this protective effect was similarly replicated in Class 3. According to Self-Determination Theory (Ryan and Deci, 2000), students who were reassigned lack intrinsic motivation for their profession and, during clinical placements, are more prone to over-alertness and exaggerated processing of negative environmental cues, which may be associated with a lack of perceived professional meaning and a higher likelihood of being categorised as extremely sensitive (Chen et al., 2025). In contrast, students with proactive motivation for their professional choice may draw on the cognitive anchoring of their career goals, potentially directing high sensitivity into professional strengths in clinical observation and empathy (Messineo et al., 2019). This suggests that interventions for the group of students reassigned to nursing should be implemented before the commencement of clinical placements, using career value clarification and motivational restructuring courses to alter their cognitive framework regarding the nursing profession.

It is worth noting that professional identity constitutes a risk factor for both Class 1 and Class 3 compared to Class 2, suggesting that professional identity has a “double-edged sword effect” on the psychological adaptation of nursing students (Lin et al., 2025). For Class 1, their strong identification with nursing is associated with high self-expectations; however, when they encounter setbacks in clinical practice, the gap between their ideals and reality may be amplified alongside heightened negative emotions in those with sensitive personalities. This aligns with the view put forward in Social Comparison Theory that “negative emotions are easily amplified when self-evaluation is threatened” (Collins, 1996). For Class 3, their strong professional identification manifests as a highly goal-oriented and pragmatic approach to work. Whilst this style aids task completion, it may inadvertently limit their capacity to attend to their own and others’ emotions (Juniarta and Sitanggang, 2024). However, it maintains stable performance in the short term, potentially leading to the overlooking of subtle changes in patients’ emotional states.

Furthermore, general self-efficacy is the most significant risk factor for Class 3. According to Bandura’s theory of self-efficacy (Bandura, 1977), anticipatory cognitive appraisals associated with high self-efficacy tend to frame clinical challenges as “controllable,” which may be associated with reduced subjective drive to process environmental stimuli in depth (Bandura, 1977). Whilst this is associated with calm and efficient task execution, it may also mask underlying deficits in empathy. These findings suggest that the influence of professional identity and self-efficacy on nursing students’ psychological adaptation is not a simple ‘the higher the better’ protective effect, but rather involves complex interactions with factors such as individual sensitivity to the environment (Miao et al., 2025).

In terms of demographic characteristics, being male, holding a bachelor’s degree, and having an urban background were protective factors associated with Class 1, suggesting that female nursing students, those without a bachelor’s degree, and those from rural backgrounds were more likely to exhibit highly sensitive traits. Gender differences are consistent with previous findings that the proportion of women with Highly Sensitive Person (HSP) traits is higher than that of men (Trå et al., 2022); in clinical nursing settings, the combined effect of high sensitivity and the demands of feminised emotional labour may further exacerbate the risk of psychological exhaustion (Feng et al., 2024). Undergraduates exhibit a lower risk of high sensitivity, which may be related to more systematic training in emotional regulation and stress management within undergraduate curricula (Balideh et al., 2025); students from urban backgrounds may possess better cognitive adaptive reserves due to richer social stimulation in their upbringing, whilst the culture shock experienced by rural students upon entering complex clinical environments should not be overlooked (Tang et al., 2025). Furthermore, a monthly household income of 3,000–5,000 yuan and less than 3,000 yuan are risk factors for Class 1 and Class 3 respectively, suggesting that economic pressure exerts differential effects on different perceptual patterns; chronic stress in low-to-middle-income backgrounds may reinforce high sensitivity traits (Haushofer and Fehr, 2014), whilst extremely low-income backgrounds may drive individuals to prioritise instrumental goals at the expense of emotional perceptual resources (Mani et al., 2013). This finding further highlights the intersection between environmental sensitivity and socioeconomic vulnerability. Students from lower-income families may face persistent financial pressure, fewer educational support resources, and stronger expectations for upward social mobility through professional success (Smith et al., 2026). In this study, a monthly per capita family income below 3,000 yuan was associated with membership in the Low Sensitive-Rational profile rather than the Highly Sensitive-Reflective profile. This suggests that socioeconomic pressure may not only increase psychological vulnerability in a direct manner but may also shape a more instrumental and goal-oriented response style. For these students, clinical failure may be interpreted less as an emotional event and more as a potential threat to employment prospects, family expectations, and future social mobility (Zhou et al., 2025). Therefore, family economic background should not be regarded merely as a demographic variable but as an important contextual factor shaping how nursing students experience environmental demands and interpret failure during clinical placements. These findings suggest that nursing education should incorporate gender, educational attainment, place of origin, and family economic background into a composite indicator system for pre-placement psychological risk screening, thereby enabling early identification of high-risk individuals and targeted interventions.

### 4.5 Preliminary practice implications for nursing education and clinical supervision

The present findings offer preliminary directional insights across three domains, sensitivity-based stratification, profile-targeted mentoring, and institutional support. Given the cross-sectional design of this study, the following suggestions are presented as provisional proposals requiring confirmation through longitudinal or interventional research before clinical adoption.

Firstly, future research could explore the feasibility and predictive validity of integrating sensitivity-based screening into pre-placement assessment frameworks. The present regression findings suggest that multidimensional indicators, encompassing professional entry motivation, gender, educational attainment, place of origin, and family economic background, may usefully inform risk stratification; sharing profile information with supervisory teams to enable tailored support planning represents a potentially viable direction (Ponce-Valencia et al., 2022). For students reassigned to the nursing programme, pre-placement career values counselling, potentially employing narrative case analysis and role-play, represents a theoretically grounded avenue for reconstructing professional meaning and attenuating vulnerability to negative clinical stimuli amplification; this warrants evaluation through controlled designs (Xue et al., 2023).

Secondly, profile-targeted mentoring represents a conceptually motivated direction for future study, with the network topology patterns serving as a preliminary, hypothesis-generating reference point; a dedicated training programme for Class 1 warrants investigation once longitudinal or experimental designs are available. For Class 1, whose network topology suggests heightened emotional reactivity and antagonistic coupling between failure mindset components, prompt one-to-one debriefings by supervising tutors following failure incidents may support adaptive attribution analysis, a practice worth incorporating into future intervention protocols. Pre-placement clinical simulation training, providing structured, graded exposure to common clinical stressors within a controlled environment including procedural error scenarios, sensory overload conditions, and critical supervisor feedback, may represent a feasible desensitisation approach targeting the S3-S4 amplification pathway in this subgroup, warranting evaluation in future experimental designs (González-Sanz et al., 2026). Complementing this, structured post-shift debriefing sessions focused on normalising and contextualising clinical errors may offer a low-threat setting for adaptive reprocessing of failure experiences, potentially reducing the likelihood of negative failure attributions consolidating into the antagonistic N1-N2 coupling pattern that characterises Class 1 (González-Tejerina et al., 2025). Mindfulness-based approaches, such as MBSR, have demonstrated efficacy in analogous clinical training populations and represent a theoretically motivated candidate for future interventional research in this subgroup, given the antagonistic coupling structure identified in the Class 1 network (Liu et al., 2024); CBT-informed attribution reframing and growth-mindset-based group programmes similarly represent theoretically aligned options for future experimental testing (Lewis et al., 2024). For Class 2, whose profile suggests heightened vulnerability to cumulative cognitive resource depletion, longitudinal monitoring of perceived stress—with adaptive supervisory pacing in response to emerging depletion signals—represents a direction worth investigating in future prospective designs (Cohen et al., 1983). For Class 3, whose network topology shows overstimulation sensitivity (S3) as the most connected node, suggesting a potential risk of insufficient perceptual depth, future research could investigate whether integrating structured attentiveness tasks into clinical supervision—such as guided observation of patient emotional cues during routine procedures—might cultivate active noticing of subtle patient signals (Xiao et al., 2025).

Finally, at the institutional level, embedding knowledge of environmental sensitivity into supervisor training programmes represents a potentially viable systemic measure (Falkenstein et al., 2025), enabling them to recognise emotional warning signs in highly vulnerable students and to master supportive communication techniques. Periodic reassessment of sensitivity profiles and failure-mindset trajectories during placement may enable timely identification of students showing deterioration who may benefit from psychological support referral; however, optimal monitoring protocols, referral thresholds, and support modalities require prospective validation (Zhang et al., 2025). The implementation of these directions would necessitate collaborative engagement among clinical supervisors, nursing education administrators, and hospital management. Collectively, the above are presented as preliminary proposals grounded in cross-sectional associational evidence; their clinical utility must be confirmed through well-designed interventional or longitudinal studies prior to adoption in practice.

## 5 Limitations

Firstly, as the study employed a cross-sectional design, it could only reflect the environmental sensitivity and failure mindset of nursing students at a single point in time; it could not infer causal relationships or dynamic changes between the two. Future research could adopt a longitudinal design, with measurements taken at multiple time points before, during, and after the placement, to verify the stability of different categories and the developmental trajectory of the failure mindset. Secondly, the sample presents two interrelated representativeness concerns. In terms of geographical coverage, participants were predominantly drawn from provinces such as Zhejiang, Jiangsu, Sichuan, and Fujian, limiting its geographical representativeness. Differences in the allocation of healthcare resources and nursing education systems across regions may affect the generalisability of the findings to other provinces or to institutions such as primary care and specialist hospitals. Future research could expand the sample coverage to explore the moderating effects of regional and cultural contexts. In terms of gender balance, the sample was markedly skewed toward female participants, which limits the generalisability of the findings in several respects. The three-profile solution may predominantly reflect female patterns of environmental sensitivity, given that women consistently score higher on Sensory Processing Sensitivity measures and that being male was the strongest protective factor against Class 1 membership in the regression. Furthermore, although sequential multi-group CFA demonstrated metric invariance across sex, the present study did not conduct gender-stratified LPA, leaving open whether the identified profiles represent gender-neutral phenotypes. The centrality of emotional reactivity as the core network node in Class 1 may similarly reflect a female-specific expression of high sensitivity rather than a universal feature. Future research should recruit more gender-balanced samples and conduct parallel multi-group LPA to verify the replicability of the three-profile taxonomy across gender before extending any intervention recommendations to male nursing students on clinical placement. Thirdly, as all variables were collected via self-report questionnaires, they may be subject to social desirability bias and common method bias (CMB); although both the Harman one-way test and the CFA-based ULCM approach indicated no severe CMB, its influence cannot be entirely ruled out. In future studies, data triangulation could be achieved by combining multiple assessment methods, such as evaluations by clinical supervisors, practical examination results, and physiological indicators. Fourthly, this study utilised the College Student Environmental Sensitivity Scale (CSESS), newly developed by Shan et al. (2025). In 2025, to measure environmental sensitivity. As this scale has only recently been published, its cross-sample validity and applicability across different populations require further validation through independent research; At the same time, as the original CSESS sample consisted of ordinary university students, the stability of the scale’s content validity and construct validity still requires careful assessment when applied to nursing students, a group characterised by a high degree of professionalisation. Future research could employ cross-validation across multiple scales to further examine the robustness of this study’s findings. Furthermore, because the network analysis is based on cross-sectional partial correlation estimates, it reveals conditional associations between variables and should not be interpreted directly as causal relationships. In particular, the Class 1 sample size is relatively small (*n* = 145), resulting in limited precision in estimating centrality indices; therefore, interpretations should be made with caution. In future studies, the sample size could be expanded, or ecological snapshot methods such as empirical dynamic assessment could be incorporated further to validate the network’s stability and causal interpretability. Additionally, the profile labels adopted in this study, notably “Reflective” for Class 1 and “Rational” for Class 3, represent theoretically informed interpretations of the observed CSESS subscale configurations rather than directly measured psychological characteristics. The present study did not include independent measures of cognitive style that would allow empirical validation of these descriptors. Future research should incorporate such measures to directly test whether the reflective and rational tendencies implied by these labels are indeed characteristic of the respective profiles, thereby strengthening the construct validity of the typology. Finally, centrality indices derived from cross-sectional networks reflect statistical connectivity patterns rather than causal mechanisms, and should not be interpreted as evidence of mechanistic priority. The present centrality-based findings are therefore preliminary and descriptive, and warrant confirmation through longitudinal or experimental designs. The relatively small Class 1 sample further limits the precision of centrality estimates in that subgroup.

## 6 Conclusion

Three distinct environmental sensitivity profiles were identified among nursing students on clinical placement, exhibiting meaningfully graded differences in failure mindset. Profile-specific network structures revealed that emotional reactivity emerged as the most central node within the highly sensitive subgroup, with negative and positive failure mindsets showing a strong inverse association; within the low-sensitivity subgroup, these two components were statistically independent. Both patterns are descriptive rather than causal and await longitudinal or experimental confirmation. Professional entry motivation, occupational identity, general self-efficacy, and selected demographic characteristics were associated with profile membership. These findings provide preliminary associational evidence consistent with the differential susceptibility framework, advancing understanding of environmental sensitivity as a heterogeneous, multidimensional construct in nursing students on clinical placement. The identified typology offers a directional foundation for future research into stratified support approaches during clinical placement; the clinical utility of such approaches, however, requires prospective validation through longitudinal or controlled interventional studies before adoption in practice.

## Data Availability

The raw data supporting the conclusions of this article will be made available by the authors, without undue reservation.
