# Supplementary material for: Profiles of environmental sensitivity among nursing students on clinical placement and their structural associations with failure mindsets: a latent profile and network analysis
Source: Front Psychol. 2026 Aug 12;17:1865123. doi: 10.3389/fpsyg.2026.1865123 (PMC13506709; doi:10.3389/fpsyg.2026.1865123)
Supplement: Supplementary file 1 [file Supplementary_file_1.DOCX]

| **Table S1:** Node centrality indices across three network structures | | | | | | | | | | | | |
| --- | --- | --- | --- | --- | --- | --- | --- | --- | --- | --- | --- | --- |
| Variable | Network 1 | | | | Network 2 | | | | Network 3 | | | |
|  | Betweenness | Closeness | Strength | EI | Betweenness | Closeness | Strength | EI | Betweenness | Closeness | Strength | EI |
| N1 | -0.848 | -0.630 | 0.311 | -1.128 | -1.118 | -1.781 | -1.770 | -1.481 | -0.598 | -0.976 | -1.150 | -1.395 |
| N2 | 1.187 | 0.921 | 0.765 | -1.227 | 0.000 | -0.164 | -0.112 | -0.398 | -0.598 | -0.716 | -0.863 | -0.164 |
| S1 | 0.170 | -0.636 | -0.640 | 0.652 | 1.118 | 0.555 | 0.178 | 0.665 | 1.793 | 1.219 | 0.769 | 0.372 |
| S2 | -0.848 | -1.334 | -1.725 | -0.145 | 0.000 | -0.251 | -0.017 | -0.331 | -0.598 | -0.812 | -0.605 | -0.852 |
| S3 | -0.848 | 0.556 | 0.417 | 0.738 | -1.118 | 0.617 | 0.448 | 0.100 | 0.598 | 1.173 | 1.289 | 1.226 |
| S4 | 1.187 | 1.123 | 0.872 | 1.109 | 1.118 | 1.024 | 1.273 | 1.445 | -0.598 | 0.113 | 0.561 | 0.813 |

Note: EI: Expected Influence. N1: Positive Failure Mindset; N2: Negative Failure Mindset; S1: Deep Perception; S2: Deep Processing of Information; S3: Prone to Overstimulation; S4: Highly Emotional. Values are standardised centrality scores.

| **Table S2:** Edge weight matrices for the three network structures | | | | | | | | | | | | | | | | | | |
| --- | --- | --- | --- | --- | --- | --- | --- | --- | --- | --- | --- | --- | --- | --- | --- | --- | --- | --- |
| Variable | Network 1 | | | | | | Network 2 | | | | | | Network 3 | | | | | |
|  | S1 | S2 | S3 | S4 | N1 | N2 | S1 | S2 | S3 | S4 | N1 | N2 | S1 | S2 | S3 | S4 | N1 | N2 |
| S1 | 1.000 |  |  |  |  |  | 1.000 |  |  |  |  |  | 1.000 |  |  |  |  |  |
| S2 | 0.191 | 1.000 |  |  |  |  | 0.479 | 1.000 |  |  |  |  | 0.169 | 1.000 |  |  |  |  |
| S3 | 0.297 | 0.043 | 1.000 |  |  |  | 0.227 | 0.000 | 1.000 |  |  |  | 0.508 | -0.096 | 1.000 |  |  |  |
| S4 | 0.243 | 0.044 | 0.633 | 1.000 |  |  | 0.178 | 0.064 | 0.468 | 1.000 |  |  | 0.227 | -0.054 | 0.378 | 1.000 |  |  |
| N1 | 0.077 | 0.068 | -0.096 | 0.051 | 1.000 |  | 0.017 | 0.200 | -0.105 | 0.015 | 1.000 |  | -0.157 | 0.331 | 0.000 | 0.000 | 1.000 |  |
| N2 | 0.000 | -0.192 | 0.000 | 0.211 | -0.752 | 1.000 | 0.000 | -0.119 | 0.154 | 0.392 | 0.178 | 1.000 | 0.000 | 0.000 | 0.235 | 0.339 | 0.000 | 1.000 |

Note: N1: Positive Failure Mindset; N2: Negative Failure Mindset; S1: Deep Perception; S2: Deep Processing of Information; S3: Prone to Overstimulation; S4: Highly Emotional.

| **Table S3** Network Comparison Test: Overall Structure and Global Strength Invariance Across Environmental Sensitivity Profiles | | | | | |
| --- | --- | --- | --- | --- | --- |
| Comparison | n₁ / n₂ | *M* | *P* (M) | *S* | *P(S)* |
| Profile 1 vs Profile 2 | 145 / 740 | 0.951 | < 0.001 | 0.302 | 0.348 |
| Profile 1 vs Profile 3 | 145 / 240 | 0.749 | < 0.001 | 0.342 | 0.232 |
| Profile 2 vs Profile 3 | 740 / 240 | 0.279 | 0.011 | 0.041 | 0.895 |
| Note: *M* = maximum absolute edge-weight difference (M-statistic); *S* = absolute difference in global network strength (S-statistic). Global network strength: P1 = 3.194, P2 = 2.892, P3 = 2.851. | | | | | |

| **Table S4** Edge-Specific Differences Surviving Benjamini–Hochberg Correction Across Profile Comparisons | | | | | | |
| --- | --- | --- | --- | --- | --- | --- |
| Comparison | Edge | \|Δ*w*\| | *w* (P1 or P2) | w (P2 or P3) | *P* | *P (BH)* |
| P1 vs P2 | N1-N2 | 0.951 | -0.854 | 0.097 | <0.001 | <0.001 |
|  | S1-S2 | 0.305 | 0.168 | 0.473 | 0.002 | 0.015 |
| P1 vs P3 | N1-N2 | 0.749 | -0.854 | -0.105 | <0.001 | <0.001 |
|  | S3-N2 | 0.329 | 0.048 | 0.377 | <0.001 | <0.001 |
|  | S1-N1 | 0.299 | 0.071 | 0.370 | <0.001 | <0.001 |
|  | S3-S4 | 0.282 | 0.388 | 0.106 | 0.006 | 0.022 |
|  | S3-N1 | 0.254 | 0.073 | 0.328 | 0.008 | 0.024 |
|  | S1-S3 | 0.239 | 0.206 | 0.446 | 0.013 | 0.032 |
| P2 vs P3 | S1-S3 | 0.279 | 0.167 | 0.446 | <0.001 | <0.001 |
|  | S1-S2 | 0.272 | 0.473 | 0.201 | <0.001 | <0.001 |
|  | S3-N1 | 0.239 | 0.089 | 0.328 | <0.001 | <0.001 |
|  | S1-N1 | 0.246 | 0.124 | 0.370 | 0.002 | 0.007 |
| *Note.* \|Δw\| = absolute difference in partial correlation weights between two profiles; w (P1 or P2) and w (P2 or P3) = edge weights in each profile estimated via the precision matrix method; *P*=permutation-based p-value; *P* (*BH*) = Benjamini–Hochberg false discovery rate adjusted p-value. Only edges significant after BH correction are reported. | | | | | | |
